# Krüppel-like factor 6 is a transcriptional activator of autophagy in acute liver injury

**DOI:** 10.1038/s41598-017-08680-w

**Published:** 2017-08-14

**Authors:** Svenja Sydor, Paul Manka, Jan Best, Sami Jafoui, Jan-Peter Sowa, Miguel Eugenio Zoubek, Virginia Hernandez-Gea, Francisco Javier Cubero, Julia Kälsch, Diana Vetter, Maria Isabel Fiel, Yujin Hoshida, C. Billie Bian, Leonard J. Nelson, Han Moshage, Klaas Nico Faber, Andreas Paul, Hideo A. Baba, Guido Gerken, Scott L. Friedman, Ali Canbay, Lars P. Bechmann

**Affiliations:** 1 Department of Gastroenterology and Hepatology, University Hospital Essen, Hufelandstrasse 55, 45147 Essen, Germany; 2 Department of Gastroenterology, Hepatology and Infectious Diseases, Otto-von-Guericke University, Leipziger Strasse 44, Magdeburg, Germany; 3 Regeneration and Repair, Institute of Hepatology, Division of Transplantation Immunology and Mucosal Biology, Faculty of Life Sciences and Medicine, King’s College London, Tower Wing Guy’s Hospital London, London, SE1 9RT United Kingdom; 4 Department of Medicine III, University Hospital RWTH Aachen, Pauwelsstrasse 30, 52074 Aachen, Germany; 5 Barcelona Hepatic Hemodynamic Laboratory, Liver Unit, Hospital Clinic, Institut de Investigacions Biomèdiques August Pi i Sunyer (IDIBAPS) and Centro de Investigación Biomédica en Red de Enfermedades Hepáticas y Digestivas (Ciberehd), Villarroel 170, 08036 Barcelona, Spain; 6 Department of Immunology, Complutense University School of Medicine, Avenida de Séneca 2, 28040 Madrid, Spain; 7 Department of Pathology, University Hospital of Essen, Hufelandstrasse 55, 45147 Essen, Germany; 8 Department of Surgery, University Hospital Zurich, Rämistrasse 100, 8091 Zurich, Switzerland; 9 Division of Liver Diseases, Department of Medicine and Tisch Cancer Institute, Icahn School of Medicine at Mount Sinai, 1425 Madison Ave., New York, NY 10029 USA; 10 Institute for Bio Engineering (IBioE), Human Tissue Engineering, Faraday Building, The University of Edinburgh, The King’s Buildings, Mayfield Road, Edinburgh, EH9 3JL Scotland United Kingdom; 11 Department of Gastroenterology and Hepatology, Center for Liver, Digestive, and Metabolic Diseases, University Medical Center Groningen, University of Groningen, Hanzeplein 1, 9713 GZ Groningen, The Netherlands; 12 Department of Laboratory Medicine, Center for Liver, Digestive, and Metabolic Diseases, University Medical Center Groningen, University of Groningen, Hanzeplein 1, 9713 GZ Groningen, The Netherlands; 13 Department of General- and Transplant-Surgery, University Hospital Essen, Hufelandstrasse 55, 45147 Essen, Germany

## Abstract

Krüppel-like factor 6 (KLF6) is a transcription factor and tumor suppressor. We previously identified KLF6 as mediator of hepatocyte glucose and lipid homeostasis. The loss or reduction of KLF6 is linked to the progression of hepatocellular carcinoma, but its contribution to liver regeneration and repair in acute liver injury are lacking so far. Here we explore the role of KLF6 in acute liver injury models in mice, and in patients with acute liver failure (ALF). KLF6 was induced in hepatocytes in ALF, and in both acetaminophen (APAP)- and carbon tetrachloride (CCl_4_)-treated mice. In mice with hepatocyte-specific Klf6 knockout (Delta*Klf6*), cell proliferation following partial hepatectomy (PHx) was increased compared to controls. Interestingly, key autophagic markers and mediators LC3-II, *Atg7* and *Beclin1* were reduced in Delta*Klf6* mice livers. Using luciferase assay and ChIP, KLF6 was established as a direct transcriptional activator of ATG7 and BECLIN1, but was dependent on the presence of p53. Here we show, that KLF6 expression is induced in ALF and in the regenerating liver, where it activates autophagy by transcriptional induction of *ATG7* and *BECLIN1* in a p53-dependent manner. These findings couple the activity of an important growth inhibitor in liver to the induction of autophagy in hepatocytes.

## Introduction

Krüppel-like factor 6 (KLF6) is a ubiquitously expressed zinc finger transcription factor, which contributes to cell proliferation, differentiation, cell death and signal transduction^1^. Hepatocyte expression of KLF6 regulates hepatic fatty acid and glucose metabolism via transcriptional activation of liver glucokinase and posttranscriptional regulation of the nuclear receptor peroxisome proliferator activated receptor alpha (PPARα)^2, 3^. KLF6-expression contributes to hepatic insulin resistance and the progression of non-alcoholic fatty liver disease (NAFLD) to non-alcoholic steatohepatitis (NASH) and NASH-cirrhosis^4^. KLF6 also affects peroxisome proliferator activated receptor gamma (PPARγ)-signaling in NAFLD^3, 5^. Besides their metabolic functions, PPARα and PPARγ regulate cell proliferation and apoptosis^6^. Furthermore, KLF6 has been identified as a tumor suppressor gene that is inactivated or downregulated in different cancers including prostate, colon and hepatocellular carcinomas^7, 8^. Consistent with its inhibitory effect on cell proliferation, KLF6 transactivates genes controlling cell proliferation, including p21, E-Cadherin and pituary tumor-transforming gene 1 (PTTG1)^8–14^. Despite its clear growth regulatory activity in hepatic metabolism and cancer, there are no studies evaluating the role of KLF6 in liver regeneration and hepatocyte proliferation.

Acute liver injury and acute liver failure (ALF) are rare but serious conditions leading to hepatocyte death that occur in a previously healthy organ. ALF is characterized by rapid induction of hepatocyte necro-apoptosis, leading to jaundice, hepatic encephalopathy and coagulopathy^15^. The underlying causes of ALF encompass autoimmune, viral, toxic or vascular diseases, with drug-induced liver injury and acetaminophen (APAP) poisoning as the most predominant etiologies in Western population^16, 17^. Acetaminophen is a widely used analgesic and antipyretic drug. Intake of high doses can result in ALF that is characterized by a rapid loss of liver cells and hepatic function due to enhanced production of reactive oxygen species (ROS), causing cellular stress and induction of cell death^17–19^. Specific treatment (N-acetyl cysteine (NAC)) promotes liver regeneration by compensation of hepatic cell loss and induction of proliferation of remaining cells and by the activation and potential differentiation of quiescent progenitor cells^20, 21^.

Liver regeneration is governed by a delicate interplay of cytokines, chemokines and the activation of proliferative and anti-apoptotic signaling pathways. Recent studies have identified autophagy, a conserved mechanism to recycle cellular components in cell starvation, to play a role in hepatocellular regeneration in APAP-induced ALF by reduction of cellular stress^22–24^. In this study, we aimed to investigate the role of KLF6 in liver regeneration following acute hepatocellular injury and ALF, and identified autophagy-related genes to be transcriptionally regulated by KLF6.

## Results

### KLF6 is induced in hepatocytes during acute human liver injury

**Figure 1:**
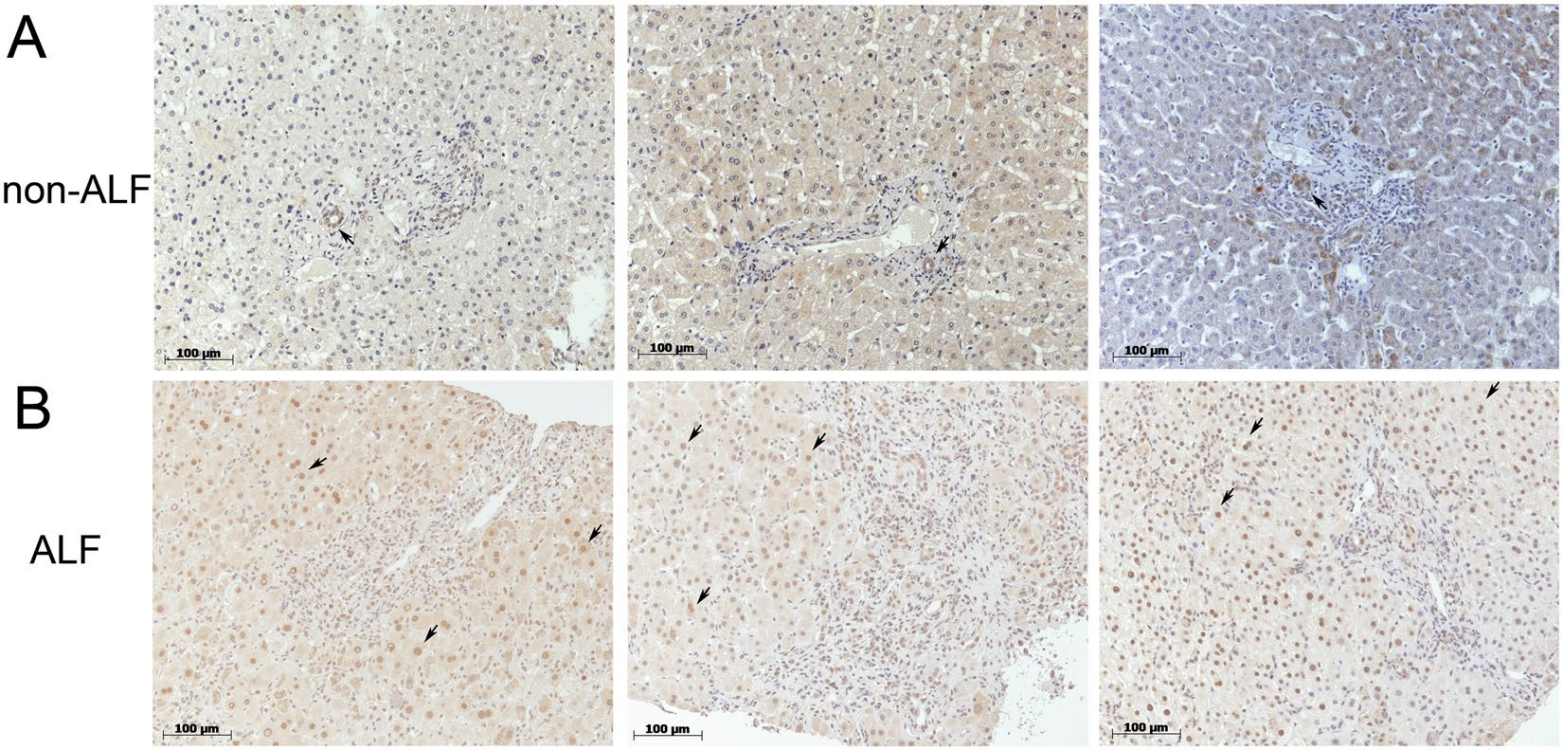
In acute liver failure (ALF) KLF6 expression is induced in hepatocytes. KLF6 protein was visualized in liver tissue of ALF patients by immunohistochemistry. Representative tissue sections of patients with drug-induced ALF (**B**) or non-acute liver injury patients (**A**) stained with KLF6 by immunohistochemistry are shown (20-fold magnification).

We compared KLF6-expression by immunohistochemistry between liver tissue from patients with ALF and without (morbidly obese patients who underwent bariatric surgery without NASH (NAS < 2) or fibrosis; for patients’ demographical data see Supplementary Table S1). KLF6-expression was low in non-acute injury livers and localized primarily in the cytoplasm of cholangiocytes, with modest staining in the cytosol or nuclei of hepatocytes (Fig. 1A). In contrast, significantly higher nuclear KLF6-expression was detected in hepatocytes in liver tissue of ALF patients, while the bile duct regions showed low levels of KLF6 (Fig. 1B; for H&E images of patients’ liver tissue, please see Supplementary Figure S1, for quantification of nuclear KLF6 in hepatocytes see Supplementary Table S1).

### KLF6 attenuates liver regeneration and autophagy after partial hepatectomy in mice

**Figure 2:**
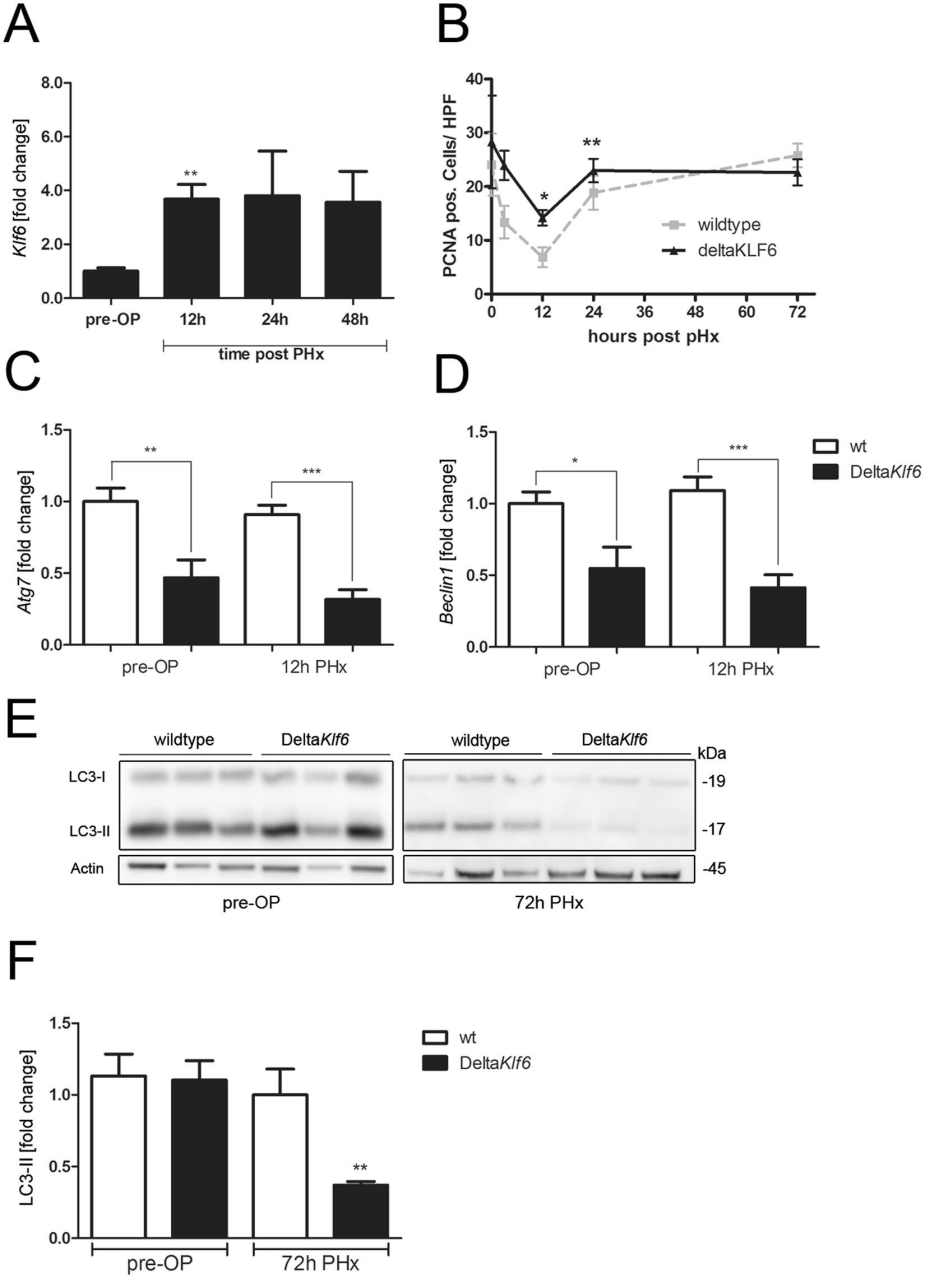
KLF6 affects liver regeneration and expression levels of autophagy-related genes after partial hepatectomy (PHx). *Klf6* expression levels were determined by qRT-PCR in mouse liver tissue before (pre-OP) and 12 h, 24 h or 48 h after 70% partial hepatectomy (PHx, n = 6/group) (**A**). Cell proliferation was assessed by quantification of PCNA positive cells in liver tissue of wildtype (wt) and Delta*Klf6* mice 72 h after PHx (**B**). Expression-levels of *Atg7* (**C**) and *Beclin1* (**D**) were measured by qRT-PCR in liver tissue of wt and Delta*Klf6* mice before and 12 h after PHx. Autophagy was assessed by LC3 Western blotting and quantified by densitometry of specific protein bands (**E**,**F**) in liver tissue of wt and Delta*Klf6* mice 72 h after PHx. Shown are representative Western blot images (**E**) and densitometric quantification of LC3-II-bands normalized to loading control beta-Actin (**F**); fold change versus control shown as mean ± SEM of n = 6 mice per group; full length Western blot images are given in Supplementary information). *Represents p-value < 0.05 and **Indicates p-value < 0.01 as assessed by 2-way ANOVA comparing wt mice with Delta*Klf6* animals at the same time point after PHx.

We performed 70% partial hepatectomy (PHx) in C57Bl/6-mice as an established model of liver regeneration^25^. Animals were sacrificed 12 h, 24 h and 48 h after PHx and the remnant liver was analyzed. Expression of *Klf6* was significantly upregulated in liver tissue following PHx in wildtype mice (Fig. 2A) and, as observed in human ALF, was mostly detected in the nuclei of hepatocytes (Supplementary Figure S2A). Next, PHx was performed in mice with a hepatocyte specific *Klf6*-knockout (Delta*Klf6*) compared to controls^2, 3^. Enhanced hepatocyte proliferation was observed in the absence of Klf6, as assessed by PCNA-staining (Fig. 2B for quantification of PCNA positive cells and Supplementary Figure S2B for immunohistochemical PCNA images), and by quantifying liver-to-body-weight-ratios (Table 1). In *KLF6*-over-expressing HepG2 cells, following transient transfection with a *KLF6*-expression vector (pcIneo-KLF6), proliferation appeared attenuated compared to empty control vector (pcIneo) transfected control cells as assessed by BrdU cell proliferation assay (Supplementary Figure S2C).

**Table 1:** Data from 12 h, 24 h or 72 h after partial hepatectomy (PHx) in wildtype or Delta*Klf6* mice.

Time post PHx	Wildtype (C57Bl/6)			Delta*Klf6*		
	12 h	24 h	72 h	12 h	24 h	72 h
ALT [U/l]	899.0 ± 649.2	315.8 ± 65.9	20.4 ± 4.4	2015.3 ± 357.1	395.0 ± 244.1	14.0 ± 3.6
AST [U/l]	2093.0 ± 513.3	753.0 ± 98.6	94.2 ± 2.04	3321.0 ± 645.6	462.5 ± 98.6	73.0 ± 10.0
Total bilirubin [mg/dl]	0.37 ± 0.12	0.45 ± 0.15	0.3 ± 0.0	0.23 ± 0.042	0.3 ± 0.0	0.23 ± 0.07
Liver/bodyweight ratio [%]	1.8 ± 0.09	1.75 ± 0.31	2.5 ± 0.19	2.28 ± 0.009**	2.5 ± 0.23**	2.1 ± 0.18

**p < 0.01 (wt vs. Delta*Klf6* at same time-point).

Liver tissue from Delta*Klf6* mice 12 h after PHx was subjected to RNA-microarray analysis (Affymetrix GeneChip HT MG-430 PM), which revealed changes in expression of autophagy-related genes compared to wt controls (heatmap included in Supplementary Figure S2D). QRT-PCR confirmed significant reductions in *Atg7* and *Beclin1* expression in Delta*Klf6* livers before and 12 h after PHx compared to wt controls (Fig. 2C,D). Attenuation of LC3-II-expression after PHx assessed by Western blot was correlated with loss of Klf6 in Delta*Klf6* mice (Fig. 2E,F). Western blot revealed high levels of LC3-II in livers of wt mice 72 h post PHx, which was significantly attenuated in the absence of Klf6 in liver tissue of Delta*Klf6* animals.

### KLF6 induction parallels induction of autophagy *in vivo* and in cell culture

**Figure 3:**
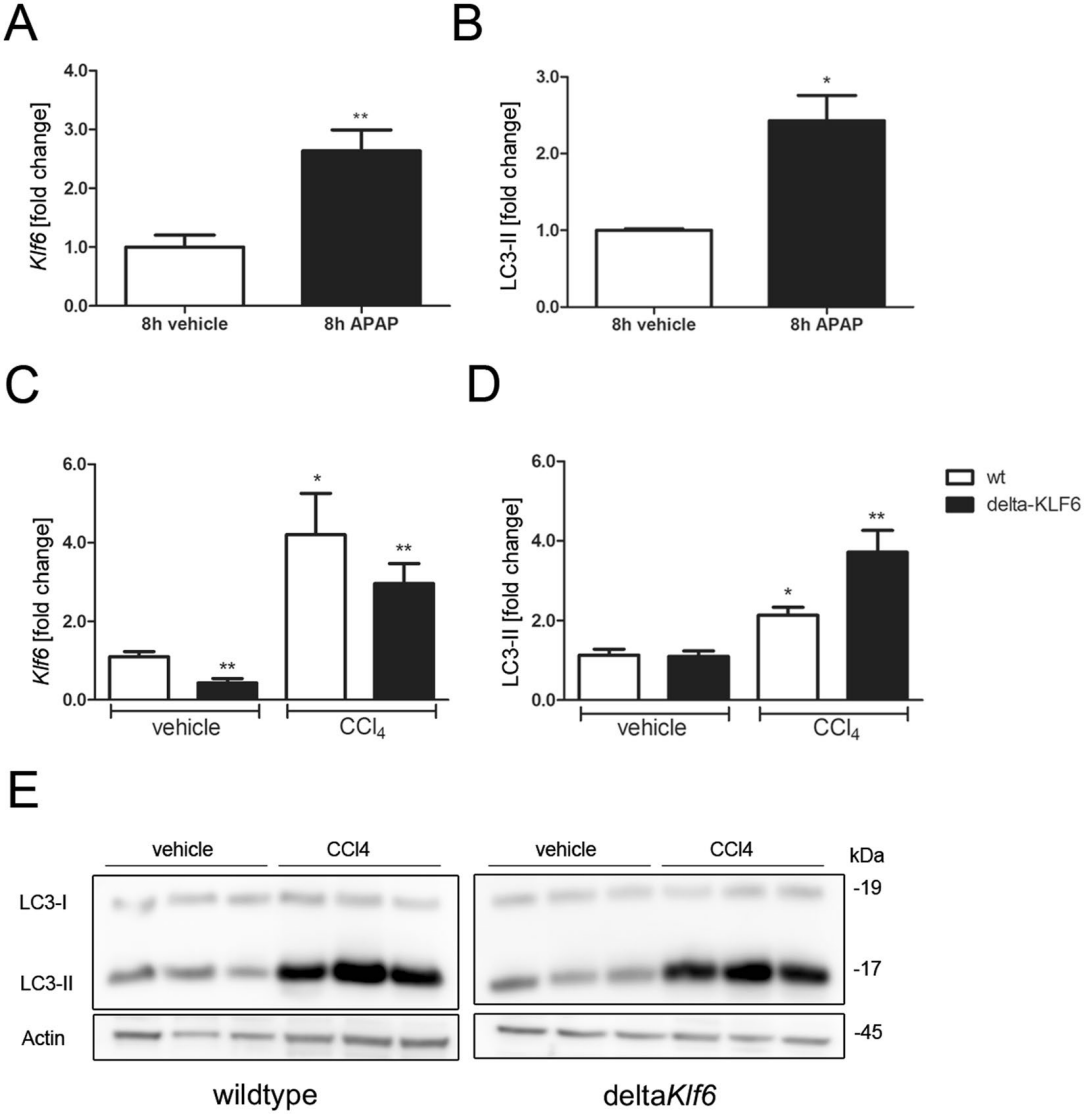
KLF6 is induced in different experimental models of acute hepatocellular injury. *Klf6*-expression levels were quantified by qRT-PCR in liver tissue of mice that received vehicle or APAP-injection (**A**; 500 mg/kg bodyweight after 8 h, n = 6) and in mice that received a single dose of CCl_4_ (**C**; 2 µl/g bodyweight, n = 4 for vehicle control and n = 5 for CCl_4_ treated animals). Autophagy was assessed by LC3 Western blotting and quantified by densitometry of specific protein bands in liver tissue of wildtype mice following APAP-injection (**B**; Western blot images of APAP-treated mice are show in Supplementary Figure S3) or in wildtype and delta*Klf*6 animals after CCl_4_ injection (**D**,**E**). Shown are representative Western blot images of CCl_4_ treated animals (**E**; full length Western blot images are given in Supplementary information) and densitometric quantification of LC3-II bands normalized to loading control Actin (**E**); fold change versus control shown as mean ± SEM of n = 4–6 mice).

To investigate *Klf6*-expression in an established *in vivo* model of APAP-induced liver injury^26^ we employed C57Bl/6-mice that received an intra-peritoneal injection of APAP (500 mg/kg bodyweight) or saline in controls (H&E images of liver tissue from APAP- or vehicle-treated mice are shown in Supplementary Figure S3A). The animals were sacrificed 8 h after injection and levels of *Klf6* gene expression in liver tissue were assessed by qRT-PCR. APAP-injection resulted in significant liver damage as indicated by increased serum ALT-, AST- and GLDH- levels 8 h after treatment (Table 2). Hepatic *Klf6*-expression was significantly increased 8 h after APAP-injection (Fig. 3A). Comparing vehicle treated C57Bl/6 mice with those receiving APAP injection; LC3-II-levels were significantly enhanced in murine liver tissue after APAP-injection (Fig. 3B, Supplementary Figure S3B for Western blot image). To evaluate *Klf6* expression in another model of acute liver injury, we injected a single dose of CCl_4_ to Delta*Klf6* and wildtype animals.In livers of animals sacrificed 48 h after receiving an acute dose of CCl_4_, in parallel to acute liver damage (see Table 2 for serum parameters of liver injury) *Klf6*-expression was as well significantly upregulated, compared to mice treated with corn oil alone (Fig. 3C). In this model of acute injury LC3-II levels were induced after CCl_4_ injection, (Fig. 3D,E).

**Table 2:** Mouse baseline parameters after treatment with APAP (wildtype animals) or acute CCl_4_ treatment (wildtype and Delta*Klf6* animals).

APAP (500 mg/kg)	Vehicle (n = 6)		APAP (n = 8)	
ALT [U/l]	53.33 ± 3.33		2259 ± 458.2**	
AST [U/l]	60.0 ± 7.07		4090 ± 720.1***	
GLDH [U/l]	10.67 ± 0.67		215.5 ± 42.62**	
Liver/bodyweight-ratio [%]	7.63 ± 0.38		8.06 ± 0.53	
**CCl**_**4**_ **(2 µl/g)**	**Vehicle (n = 4)**		**CCl**_**4**_ **(n = 5**)	
	**wt**	**Delta** ***Klf6***	**wt**	**Delta** ***Klf6***
ALT [U/l]	38.3 ± 8.4	49.2 ± 3.4	733.2 ± 105.7**	704 ± 132.6**
AST [U/l]	12.8 ± 0.8	6.0 ± 1.6**	1444 ± 198**	1423 ± 234.5**
Total bilirubin [mg/dl]	0.1 ± 0.0	0.1 ± 0.0	0.26 ± 0.04*	0.25 ± 0.05*

*p < 0.05; **p < 0.01; ***p < 0.001 versus vehicle control.

To validate the *in vivo* observations, we quantified *KLF6*-expression in APAP treated cell culture models. Therefore, we treated HepaRG cells, which resemble the metabolic function of human hepatocytes, with different concentrations of APAP (5 mM, 10 mM and 20 mM) for 24 h to induce cellular stress and damage. Here, *KLF6* was significantly upregulated after APAP treatment in a dose-dependent fashion (Supplementary Figure S3C). Similarly, in HepG2 cells, treatment with APAP for 24 h significantly induced *KLF6*-levels (Supplementary Figure S3D). We then quantified autophagy-induction in APAP-treated HepG2 cells and observed increased LC3-II and p62 levels compared to control cells (Supplementary Figure S3E,F,H and I).

### KLF6 induces autophagy and binds to promoter regions of BECLIN1 and ATG7

**Figure 4:**
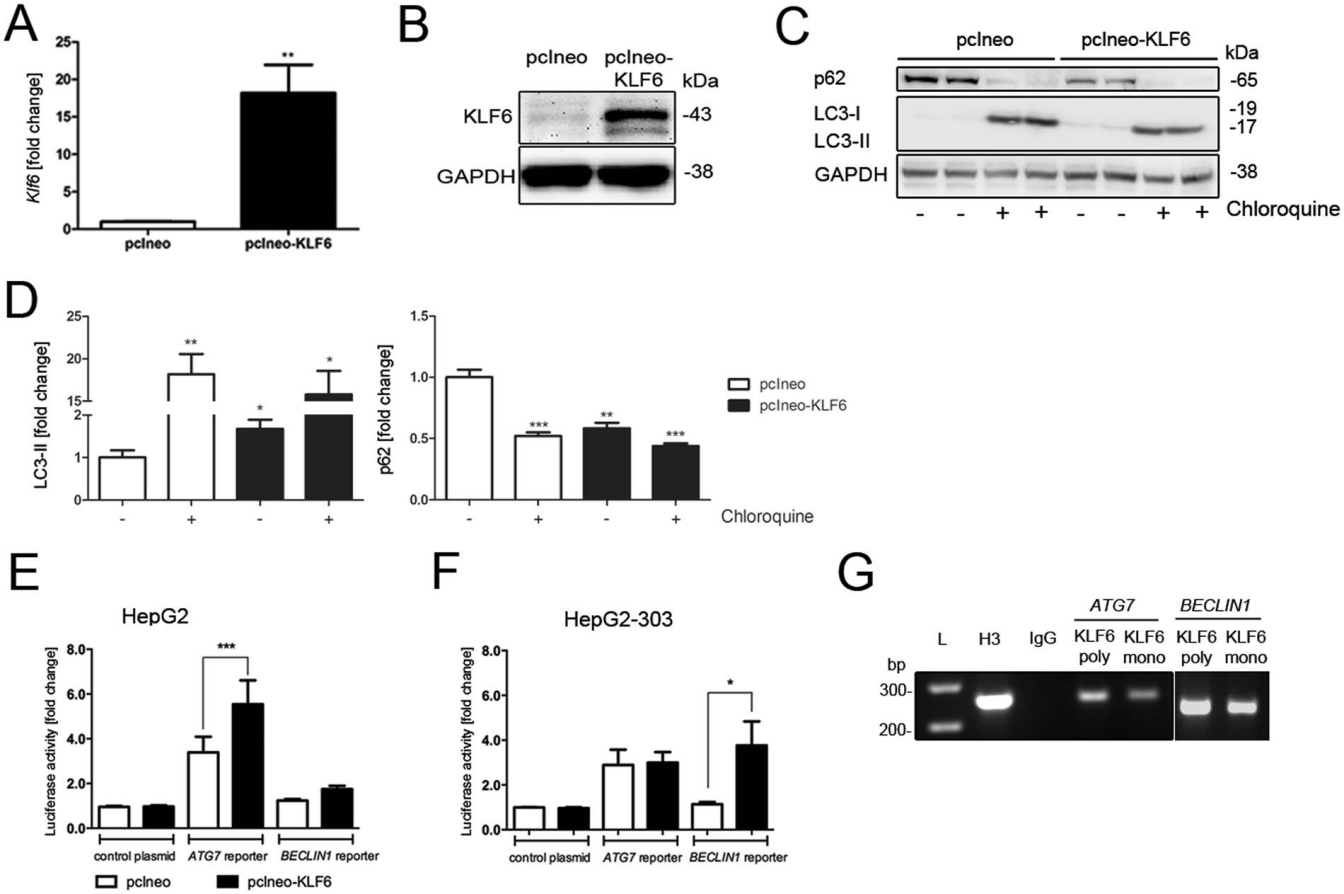
KLF6 transcriptionally activates autophagy-related genes in a p53-dependent manner. Transfection with the *KLF6*-expression vector pcIneo-KLF6 induced KLF6 mRNA (**A**) and protein (**B**) expression. KLF6-over-expression induces autophagy in HepG2 cells as assessed by LC3 Western blotting (**C**) and quantification of LC3-II-bands normalized to loading control GAPDH (**D**; fold change versus control shown as mean ± SEM of n = 3 independent cell culture experiments; full length Western blot images are given in Supplementary information.). Autophagic flux was assessed by LC3-II and p62 Western blotting in HepG2 and pcIneo-KLF6 transfected cells in absence (−) or presence ( + ) of 100 µM chloroquine for 24 h ((**C**) shows representative Western blot images, (**D)** shows fold change versus control, shown as mean ± SEM of n = 3 independent experiments). Activation of the *ATG7* and *BECLIN1* promoter was quantified by luciferase activity in a co-transfection experiment of pcIneo-KLF6 with specific promoter reporter luciferase plasmids in HepG2 (**E**) and in p53-deficient HepG2-303 cells (**F**). The interaction of KLF6 with the *ATG7* and *BECLIN1* promoter containing putative KLF6-binding sites was confirmed by chromosomal immunoprecipitation (ChIP) in HepG2 cells (**G**) using two different KLF6 antibodies, IgG as negative controls, Histone-H3 antibody was used as a positive control for ChIP; full length images of agarose gels are given in Supplementary information.

**Figure 5:**
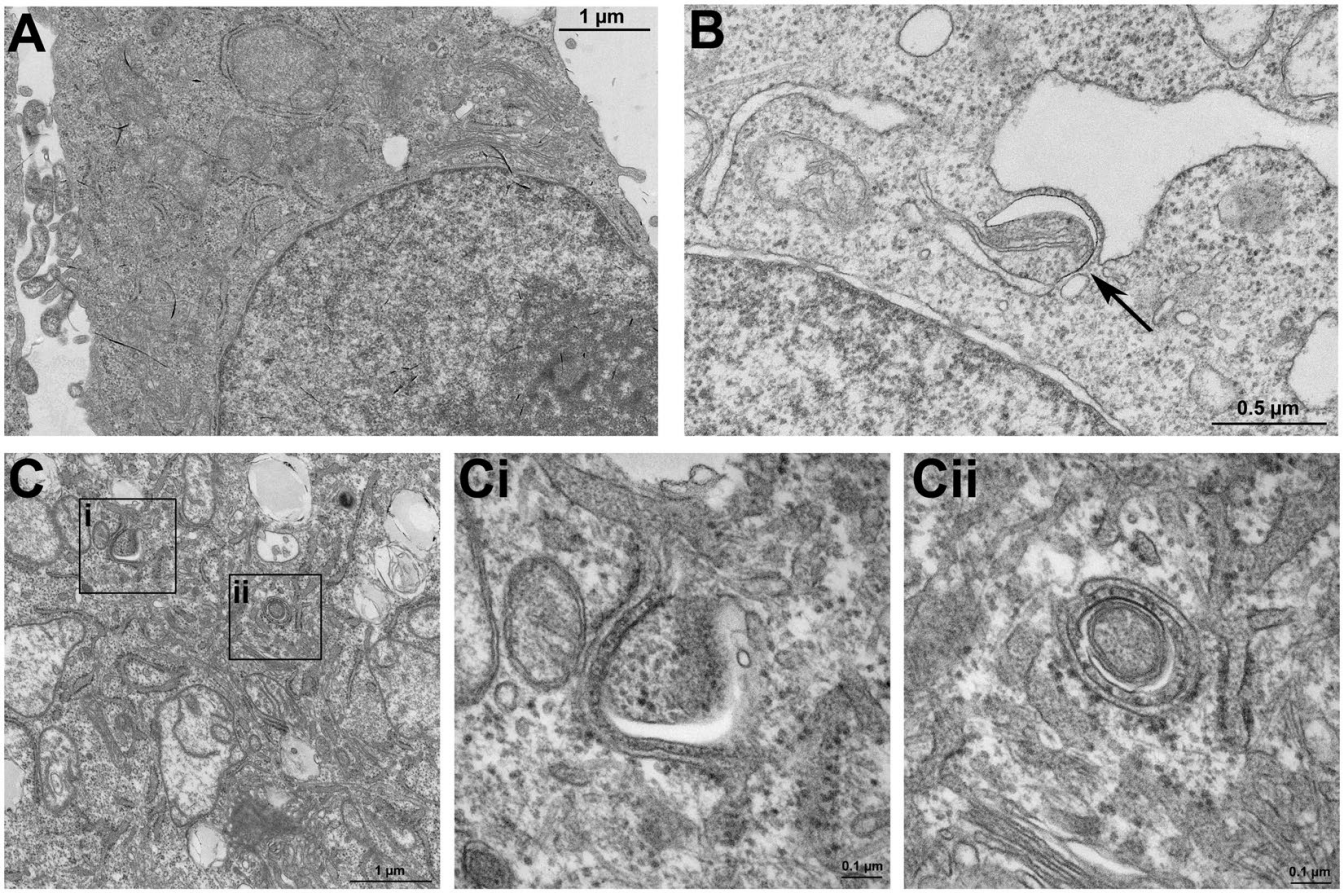
Autophagosome formation in *KLF6* over-expressing HepG2 cells. Under a transmission electron microscope, the autophagosome formation was observed and imaged in HepG2 cells transfected with the empty vector (pcIneo) (**A**), in HepG2 cells transfected with pcIneo treated with 15 µM rapamycin for 6 h to stimulate autophagosome formation (**B**) and in *KLF6*-over-expressing HepG2 cells (pcIneo-KLF6) (**C**). Representative slides and blow-ups shown are shown of n = 2 independent cell culture experiments.

**Figure 6:**
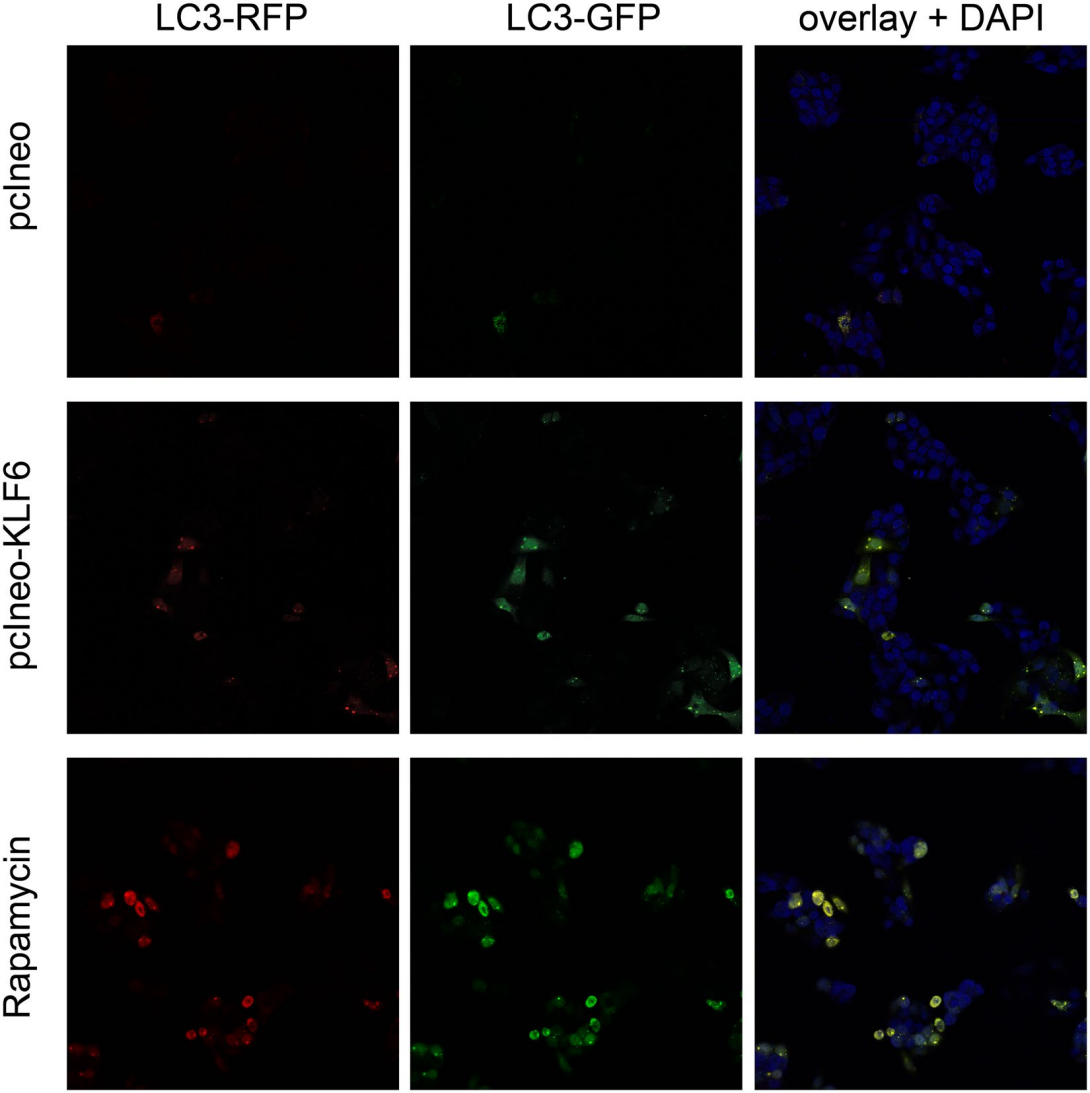
Autophagosome formation in KLF6 over-expressing HepG2 cells. To visualize formation of autophagosomes in HepG2 cells transfected with empty control vector (pcIneo) or in KLF6 over-expressing HepG2 cells (pcIneoKLF6) we treated the cells with Autophagy Tandem Sensor RFP-GFP-LC3B. As a positive control, we stimulated autophagosome formation via treatment with 15 µM rapamycin for 6 h. Cells were viewed and imaged with a Leica SP8 confocal microscope (20-fold magnification); shown are representative images of n = 3 individual cell culture experiments.

To verify the functional interaction between KLF6 and autophagy related targets, we transiently transfected HepG2 cells with an empty control vector (pcIneo) or a KLF6-expression vector (pcIneo-KLF6) in order to quantify autophagy induction in KLF6-over-expressing cells (Fig. 4). In parallel to KLF6-overexpression (Fig. 4A,B), LC3-II was increased and p62 levels were decreased in these cells (Fig. 4C,D). To assess autophagosome formation, we performed transmission electron microscopy with control vector transfected HepG2 (pcIneo) cells and pcIneo-KLF6 transfected HepG2 cells. In *KLF6* over-expressing HepG2 cells (Fig. 5C), there were more autophagy-positive cells compared to pcIneo-transfected HepG2 cells (Fig. 5A; for quantification of autophagy-positive cells see Supplementary Table S2). As a control for autophagy induction and autophagosome-formation we treated pcIneo-transfected HepG2 cells with 15 µM of rapamycin for 6 h (Fig. 5B). In addition, we performed Autophagy Tandem Sensor RFP-GFP-LC3B assay (Fig. 6), which confirmed increased formation of autophagosomes in KLF6-over-expressing HepG2 cells (Fig. 6; Supplementary Table S2). LC3-II turnover was assessed by Western blot in HepG2 (pcIneo) and KLF6 over-expressing HepG2 cells (pcIneo-KLF6) in the absence or presence of the lysosomal inhibitor chloroquine (100 µM for 24 h; Fig. 4C,D). In the absence of the inhibitor, KLF6 over-expression led to an increase of LC3-II and a decrease of p62 in comparison to pcIneo transfected cells. Incubation with the lysosmal inhibitor chloroquine resulted in a clear accumulation of LC3-II and a suppression of p62 in both pcIneo and in pcIneo-KLF6 cells. The use of the lysosomal inhibitor chloroquine demonstrated that autophagic flux occurs in these cells. However, in this experiment, there were no significant differences in LC3-II levels following chloroquine treatment comparing pcIneo and pcIneo-KLF6 transfected HepG2 cells (Fig. 4D).

KLF6 belongs to the family of zinc finger proteins that regulate target genes and cellular pathways by binding to specific DNA motifs. We identified potential KLF6 binding motifs within the promoter regions of the autophagy related genes *ATG7* and BECLIN1, and then confirmed transcriptional activation by luciferase reporter assays. To do so, we performed co-transfection with reporter plasmids in *KLF6*-over-expressing HepG2 cells and specific luciferase reporter plasmids carrying the promoter regions of *ATG7* or *BECLIN1*. A background control was comprised of a commercially available random control vector containing a non-conserved, non-genic and non-repetitive fragment of equal length to the specific sequence upstream of the luciferase gene. As shown in Fig. 4E in HepG2 cells over-expressing *KLF6*, *ATG7* promoter activity was significantly higher compared to control plasmid transfected cells. Thus, KLF6 transactivates *ATG7* and therefore might influence the level of autophagy. Next, we performed chromatin immune precipitation assays (ChIP), which confirmed direct binding of KLF6 to the promoter regions of *ATG7* and *BECLIN1* (Fig. 4G). Interestingly, despite active binding of KLF6 to the *BECLIN1* promoter, BECLIN1 luciferase activity was not altered by *KLF6*-overexpression in HepG2 cells (Fig. 4E). Predicted binding elements of KLF6 to promoter regions of ATG7 or BECLIN1 were assessed by using ChIP-seq data from KLF6-transfected HepG2 cells that were obtained from the NIH Encyclopedia of DNA Elements (ENCODE) database. This analysis clearly identified protein-DNA binding sides of KLF6 on regions encoding ATG7 and BECLIN1 (Supplementary Figure S2F+G).

### The p53-dependant transcriptional activation of ATG7 and BECLIN1 by KLF6 is independent of apoptosis

A direct interaction between KLF6 and p53 has previously been demonstrated in the context of IGF-1 regulation^27, 28^. In contrast to KLF6, several direct and indirect interactions between autophagy and p53 have identified p53 as an important regulator of autophagy^29^. To investigate a potential interaction between KLF6 and p53 in the context of autophagy-induction, we used p53-deficient HepG2-303 cells to determine if KLF6 still leads to upregulation of autophagy-related genes in the absence of p53. To do so, we performed luciferase assays in *KLF6*-over-expressing HepG2-303 cells transfected with promoter-reporter constructs for *ATG7* and *BECLIN1*. In contrast to p53-expressing HepG2 cells, the *ATG7* promoter was not activated in *KLF6*-over-expressing HepG2-303 cells (Fig. 4F). Interestingly, the activation of *BECLIN1* in HepG2-303 cells was enhanced in *KLF6*-over-expressing cells compared to control cells, pointing towards p53-dependent (*ATG7*) and p53-independent (*BECLIN1*) mechanisms, by which KLF6 regulates autophagy-related effector proteins. However, LC3-II-levels were obviously not changed in HepG2-303 cells treated with APAP or in HepG2-303 cells over-expressing *KLF6* (Supplementary Figure S3G,J), implying that p53 is required to enhance LC3-II as a marker for increased autophagosome formation.

To elucidate potential non-transcriptional effects of KLF6 on autophagy-induction, we further investigated its role in apoptosis-induction. Following cellular stress, autophagy can block apoptosis or caspase activation and promote survival by clearance of reactive oxygen species or damaged proteins. A switch from autophagy to apoptosis may occur, since autophagic and apoptotic molecules including BECLIN1 and BCL-2 interact directly^30, 31^. Since p53 is also an activator of apoptosis and several mediators involved in autophagy-induction also contribute to Caspase-regulation/apoptosis-regulation, we measured expression levels of the apoptosis-related molecules *BAX*, *BAD* and *BCL-2*. Expression of these genes was not changed in *KLF6*-over-expressing cells compared to empty vector transfected HepG2 cells. However, as previously published, expression levels of *P21* were reduced in *KLF6*-over-expressing HepG2 cells (Supplementary Table S3). Additionally, we performed a Proteome Profiler human Apoptosis Array to analyze the expression profiles of 35 apoptosis-related proteins using cell lysates from normal HepG2 cells (pcIneo) and *KLF6*-over-expressing HepG2 cells. This array did not highlight any differences between control vector transfected and *KLF6*-over-expressing HepG2 cells (Supplementary Figure S2E).

## Discussion

KLF6 is a growth suppressor gene and the inactivation of KLF6 is associated with multiple human tumors^7, 8, 11^. Among several mechanisms of tumor suppression, KLF6 inhibits cell cycle progression and proliferation^32^. However, the behavior of KLF6 during liver regeneration following acute liver injury has not been assessed to date. With this study we establish that KLF6 is induced and translocated to the nucleus in hepatocytes among different models of acute liver injury. This activation is associated with enhanced hepatocyte proliferation in early liver regeneration. We further identify KLF6 as a transcriptional activator of ATG7 and BECLIN1, thereby establishing KLF6 as a novel mediator of autophagy. This novel function of KLF6 depends on the presence of p53, but appears to be independent of apoptosis.

Healthy liver tissue has the ability to compensate for the loss of organ function in case of induced cell stress, acute injury or cell death. However, the excessive loss of functional liver tissue may lead to ALF. Following cell loss or death, activation of cell proliferation and regeneration, combined with attenuation of growth suppressor activity within remnant liver tissue restores liver cell mass. Downregulation of KLF6, a tumor suppressor gene that inhibits proliferation through induction of p21 and in synergy with p53^8, 10, 12^ has been observed in primary liver tumors and is associated with a worse outcome in cancer^1, 11, 33^. Following PHx in mice, hepatocyte-specific deletion of *Klf6* accelerates cell proliferation at early time points after resection. The later loss of growth induction in Delta*Klf6* mice suggests that mechanisms not related to hepatocellular Klf6 override its anti-proliferative effects as hepatocyte regeneration progresses. Furthermore, these observations might as well be confounded by Klf6-expression in non-parenchymal cells^34, 35^. Here, cell proliferation was slightly reduced in *in vitro* experiments using *KLF6*-over-expressing HepG2 cells as shown by BrdU assay. Furthermore, *KLF6*-overexpression was accompanied with reduced expression levels of *p21* in transiently transfected HepG2 cells. This transcription factor regulates cell cycle progression, DNA replication and repair by regulating the activity of different cyclin dependent kinases; its activation is controlled by the tumor suppressor protein p53^36, 37^. Nonetheless, we observed a strong hepatocyte induction of KLF6 in models of acute liver injury and ALF patients, and an early proliferative advantage for hepatocyte-specific *Klf6* knockout mice undergoing PHx.

A marked reduction of autophagic vesicles in hepatocytes was first observed in 1979 by Pfeifer in rats undergoing PHx^38^. More recently, autophagy has been established as an essential mechanism required for liver regeneration after PHx, since in liver-specific *Atg5* knockout mice liver regeneration and cell division are markedly impaired after PHx due to reduced ATP levels and decreased β-oxidation^39^. Here, utilizing a hepatocyte-specific *Klf6* knockout model, we identified *Klf6* as a transcriptional activator of the autophagy related genes *Atg7* and *Beclin1* in PHx and acute CCl_4_ induced liver injury. Metabolism of APAP results in formation of NAPQI (N-acetyl-p-benzoquinone imine), which reacts with glutathione (GSH) to form GSH-adducts that can be secreted. In APAP overdose with progressive GSH-depletion NAPQI binds to cellular proteins and causes mitochondrial damages leading to cell death (mainly necrosis) and inflammation. In liver injury following APAP overdose, autophagy represses apoptosis, reduces cellular stress, inflammation and injury by removing damaged cells and organelles^22–24, 31^. Ni *et al*. showed that SQSTM1/p62 plays an important role in reducing APAP protein adducts, while after shRNA-mediated p62-knockdown APAP protein adducts were increased in primary hepatocytes^23^. In aging mice, autophagy and hepatocellular apoptosis are induced, leading to impaired liver regeneration following PHx^40^. In a related study, autophagy played a critical role in liver regeneration and in the preservation of cellular quality, preventing hepatocytes from becoming fully senescent and hypertrophic. This effect was most likely mediated by p21 and stimulation of interleukins^39^. Interestingly, in a PHx model, mTOR inhibition severely impaired liver regeneration and increased autophagy rate. These effects were partly reversed by stimulation of the IL-6 and HGF pathways^41^.

Our gene array data uncovered altered expression of autophagy-regulatory proteins in mice lacking hepatocyte Klf6 (Delta*Klf6* mice) following PHx. Accordingly, we documented the parallel induction of autophagy and KLF6 in several models of liver injury. In Delta*Klf6* mice, autophagy-induction was attenuated compared to controls and *KLF6*-over-expressing HepG2 cells showed increased LC3-II accumulation and formation of autophagosomes, while there was no evidence for increased autophagic flux in conditions of KLF6-over-expression as compared to control conditions. We then analyzed whether KLF6 functionally interacts with promoter regions of several autophagy-related genes, which contain conserved KLF6-binding motifs. ChIP assay analysis confirmed direct binding of KLF6 to promoter regions of *ATG7* and *BECLIN1*. Interestingly, KLF6-mediated transcriptional activation of *ATG7* is dependent on p53, since *KLF6*-overexpression activated the *ATG7* promoter in HepG2, but not in p53 deficient Hep-G2-303 cells. Conversely, *BECLIN1* transcriptional activation was induced by KLF6-overexpression under p53 deficient conditions, while KLF6 had no effect on *BECLIN*1 in HepG2 cells.

A functional interaction between KLF6 and p53 has previously been described. Rubinstein *et al*. observed that a transcriptional effect of KLF6 on the IGF-1 receptor is dependent on the presence of p53^27^, and KLF6 itself is a transcriptional target of IGF1, which also requires p53^28^. KLF6 can also repress MDM2, which binds to the tumor suppressor p53 and thus accelerates its degradation in a mouse model of hepatocellular cancer^8^. Here, we observed a novel transcriptional activity of KLF6 by inducing two autophagy related genes (*BECLIN1* in p53 deficient cells and *ATG7* in the presence of p53) is switched, based on the presence or absence of p53. Beyond its role in autophagy, Beclin1 has also been described as a tumor suppressor gene in many cancer types and shares a BH3 domain with pro-apoptotic genes like Bid or Bad^42^. In our study expression levels of BAX, BID and BCL-2 were not changed in KLF6 over-expressing HepG2 cells.

Furthermore, Beclin1 can alter p53 expression by regulating deubiquitination of p53 by USP10^43^. To date, no interaction between KLF6 and either BECLIN1 or ATG7 has been reported. Interestingly, the absence or presence of p53 determines a pro-tumorigenic or tumor-suppressing property of autophagy in a mouse model of pancreatic cancer^44^. Thus, KLF6 might serve as an important mediator in autophagy-induction but has no impact on apoptosis in the context of acute liver injury.

Taken together, our findings establish that KLF6-expression is induced in models of acute liver injury and in patients with ALF. Here, we describe for the first time a direct transcriptional activation of autophagy-related genes by KLF6. This transcriptional activation depends on the presence (*ATG7*) or absence (*BECLIN1*) of p53. Thus, KLF6 drives autophagy-induction and autophagy-related cell death in acute liver injury.

## Material and Methods

### Cell culture

HepG2 cells were grown in DMEM-High-Glucose medium (Invitrogen, Calrsbad, CA, USA) with 10% of fetal bovine serum (FBS, Biochrom, Berlin, Germany, 1000 U/ml penicillin, 0.1 mg/ml streptomycin and 2 mM L-glutamine (PAA, Pasching, Austria). Cells were kept in an atmosphere with 5% CO_2_ under 37 °C following standard protocols. In HepG2-303 cells p53 was stably knocked out and they were cultivated as described elsewhere^45^. Additional cell culture protocols are provided in Supplementary material.

### Transfection conditions, reporter assay and BrdU assay

For transient transfection cells were seeded one-day prior transfection on different plate formats (6-well, 12-well, 96-well) at a density of 5 × 10^4^ cells/cm^2^. KLF6-over-expressing HepG2 cells were transfected using Transfectine (Bio-Rad, Munich, Germany) at a ratio of 3 µl Transfectine per µg DNA as recommended by the manufacturer. KLF6-expression plasmid pcIneo-KLF6^34^ or empty control vector pcIneo (Promega, Madison, WI, USA) were used at 80 ng per 3.9 cm^2^ well. For luciferase reporter assays HepG2 were co-transfected with pcIneo or pcIneo-KLF6 and 100 ng of reporter plasmid vectors pLightSwitch-*ATG7*, pLightSwitch-*BECLIN1* or pLightSwitch-random control plasmid (SwitchGear Genomincs, Menlo Park, CA, USA). Luciferase assay was performed using LightSwitch-Luciferase assay system following manufacturer’s instruction (SwitchGear Genomics). BrdU assay was performed using the Cell Proliferation ELISA BrdU Kit following manufacturer’s instructions (Roche, Mannheim, Germany).

### Transmission electron microscopy and Autophagy Tandem Sensor assay

HepG2 cells were transfected as described above with pcIneo or pcIneo-KLF6. For induction of autophagy and monitoring of autophagosome-formation, HepG2 cells were treated with 15 µM of Rapamycin for 6 h (Medchem Express, Monmouth Junctions, NJ USA). After incubation, cells were fixed for 2 h at room temperature using 2.5% glutaraldehyde in 0.1 M PB buffer (0.1 M Na_2_HPO_4_; 0.1 M KH_2_PO_4_ buffer). Cells were washed with PB buffer, removed from the cell culture dish; the cell pellet was postfixed in 2% osmium tetroxide, dehydrated in a graded series of alcohol and embedded in epoxy resin. Ultrathin sections were post-stained with uranyl acetate (1%) and lead citrate (0.4%). Sections were viewed in a Jeol TEM1400 Plus (Jeol, Tokyo, Japan). For visualization of autophagosomes in pcIneo or pcIneo-KLF6 transfected HepG2 cells we used the Premo™ Autophagy Tandem Sensor RFP-GFP-LC3B Kit (Thermo Scientific/Life Technologies, Darmstadt, Germany) according to manufacturer’s protocol. Fixed cells were viewed with a Leica SP8 confocal microscope (Leica Microsystems, Wetzlar, Germany).

### Chromatin immunoprecipitation assay

For Chromatin immunoprecipitation assay (ChIP) cells were cross-linked with a final concentration of 1% formaldehyde for 10 min at 37 °C, then washed and harvested in SDS lysis buffer (10% SDS; 0.5 M EDTA; 1 M Tris-HCl; containing proteinase inhibitor cocktail from Sigma-Aldrich, St. Louis, MO, USA) and sheared by sonication to fragment DNA. Samples were immunoprecipitated with 10 µg of anti-KLF6 antibody (polyclonal antibody KLF6 (R-173) or monoclonal antibody KLF6 (E-10) (Santa Cruz Biotechnologies, Dallas, TX, USA), anti-histone H3 antibody (Abcam, Cambridge, UK) or control IgG (Abcam) and protein-A/G agarose beads (Santa Cruz Biotechnologies). Following removal of cross-linked DNA/protein complexes by Proteinase K (Qiagen, Hilden, Germany) treatment, immunoprecipitated DNA was purified using QIAamp DNA Mini Kit (Qiagen) and used for PCR with *ATG7* or *BECLIN1* primers (Supplementary Table S3), encompassing the promoter region −200 bp to −400 bp upstream of transcriptional start site to amplify immunoprecipitated DNA, PCR products were visualized on an agarose gel.

### Animals and surgical procedures

Mice with a floxed *Klf6* targeting vector (C57Bl/6;129Sv, Genentech, San Francicso, CA, USA)^46^ were crossed with mice expressing Cre recombinase (Cre) under control of the albumin promoter (B6.Cg-Tg(Alb-cre) 21 Mgn/J; Jackson Labs, Bar Habor, ME, USA). After backcrossing, male offspring expressing Cre with two floxed *Klf6* alleles were used as the experimental group (‘Delta*Klf6*’). Mice with two floxed alleles and no Cre expression were used as controls (wt). Temperature, humidity and light-dark cycle conditions were controlled; mice were allowed food and water *ad libitum*. Before surgical intervention animals were anesthetized, 70%PHx was performed by removing the left and median lobes of the liver^47^. Mice were sacrificed after 3 h, 12 h, 24 h, 48 h and 72 h following surgical intervention, respectively. Protocol for Affymetrix microarray analysis from liver tissue post PHx is given as Supplementary material. To induce APAP-induced liver injury C57Bl/6 mice received intraperitoneal injection of APAP (500 mg/kg bodyweight, Sigma-Aldrich) and were sacrificed 8 h after APAP-administration^26^. Carbon tetrachloride (CCl_4_, Sigma-Aldrich)-induced acute liver injury was achieved by intra-peritoneal injection of 2 µl/g bodyweight of CCl_4_ or corn oil; animals were killed after 48 h. Experiments were conducted in three different facilities in accordance with relevant guidelines and regulations. Studies were approved by the Institutional Animal Care and Use Committee (IACUC) of Icahn School of Medicine at Mount Sinai (reference number LA09-00251), and the State authority for environment and animal welfare in Northrhine-Westfalia (LANUV, reference number 84-02.04-2013) for work conducted at the University of Duisburg-Essen and the RWTH Aachen. For baseline characteristics see Tables 1 and 2.

#### Ethical considerations

All investigations in human material and the use of patient samples were approved by the Ethics Committee (Institutional Review Board) of the University Hospital Essen (reference numbers: 14-6066-BO and 09-4252) and the study protocol conformed to the ethical guidelines of the Declaration of Helsinki. Sample allocation in non-acute liver injury patients that underwent bariatric surgery was undertaken following patients’ informed consent. As patient data and samples of the historic cohort of ALF patients were analyzed retrospectively from stored samples that were obtained for routine clinical use, informed consent from these subjects was explicitly not required according to the local ethics committee.

### Histopathology and sample handling

Liver tissue from mice or ALF patients (Supplementary Table S13) was stored in 4.5% formalin-solution, paraffin-embedded and sectioned. Stainings were performed using standard protocols; rabbit anti-KLF6 (R-173; Santa Cruz Biotechnology). Liver tissue for RNA and protein isolation was frozen in liquid nitrogen. Total RNA and protein from liver tissue were isolated by TRIzol® extraction (Invitrogen), RNA was purified utilizing RNeasy Mini Kit (Qiagen). Protein lysates from cells were prepared using lysis buffer (50 mM Tris-HCl; 150 nM NaCl; 0.1% NP-40; 1% desoxycholic acid) containing complete mini EDTA-free protease inhibitor cocktail and phosphostop (Roche).

### Quantitative real time PCR

Reverse transcription was performed with the QuantiTect-RT kit (Qiagen) using 1 µg of total RNA. Specific mRNA expression levels were measured by quantitative realtime-PCR (qRT-PCR) performed on a CFX96 Touch Real-Time PCR Detection System (Bio-Rad) using QuantiTect SYBR Green Kit (Qiagen) in a volume of 15 μl including 2 μl of cDNA. Oligonucleotide sequences of used primers are shown in Supplementary Table S4. Melting curves were collected to ascertain specificity of PCR-products. Changes in mRNA-expression were calculated by the ΔΔ-Ct method and are presented as foldchange in relation to expression of a reference gene (*HPRT* or *Sdha*).

### Western blotting

For SDS-PAGE 30 µg of total protein were separated; immunoblotting was performed using standard procedures with the following primary antibodies: LC3 (Abcam), KLF6-R173 (Santa Cruz Biotechnologies), p62 (Enzo Lifesciences, Antwerpen, Belgium), β-Actin 13E5 and GAPDH 14C10 (Cell Signaling). After incubation with the appropriate horseradish peroxidase-conjugated secondary antibody, bound antibodies were visualized using ECL-Prime (GE Healthcare, Chalfont St. Giles, UK). Blotting images were generated using ChemiDoc System and Quantity One software (Bio-Rad) to quantify the densities of the bands.

### Statistical analysis

Statistical significance was determined using an unpaired (or paired, when applicable), two-tailed *t*-test or by one-way ANOVA (with Tukey’s post-hoc test for individual experimental conditions) performed with GraphPad Prism 6 (GraphPad Software Inc., San Diego, CA, USA). Significance was assumed at *p* ≤ 0.05. If not stated otherwise all data are presented as mean ± SEM.

### Data availability

Array data can be found at (http://www.ncbi.nlm.nih.gov/geo/query/acc.cgi?acc=GSE85381), under the accession number GSE85381.

## Electronic supplementary material

Supplementary Information
