# Supplementary material for: Krüppel-like factor 6 is a transcriptional activator of autophagy in acute liver injury
Source: Sci Rep. 2017 Aug 14;7:8119. doi: 10.1038/s41598-017-08680-w (PMC5556119; doi:10.1038/s41598-017-08680-w)

Krüppel-like factor 6 is a transcriptional activator of autophagy in acute liver injury

Svenja Sydor, Paul Manka, Jan Best, Sami Jafoui, Jan-Peter Sowa, Miguel Eugenio Zoubek, Virginia Hernandez-Gea, Francisco Javier Cubero, Julia Kälsch, Diana Vetter, Maria Isabel Fiel, Yujin Hoshida, C. Billie Bian, Leonard J. Nelson, Han Moshage, Klaas Nico Faber, Andreas Paul, Hideo A. Baba, Guido Gerken, Scott L. Friedman, Ali Canbay and Lars P. Bechmann*

Corresponding author*:

Lars P. Bechmann, MD, MBA

Department of Gastroenterology, Hepatology and Infectious Diseases

Leipziger Strasse 44

D-39120 Magdeburg

Germany

Mail: lars.bechmann@med.ovgu.de

Phone: +49-391-67-21522

Fax: +49-391-67-13105

Supplementary data: Supplementary methods, 3 Supplementary Figures, and 4 Supplementary Tables

Supplementary methods:

Microarray

RNA for expression analysis on microarrays was processed as described before [1]. Briefly, RNA samples were quantified on a NanoDrop 1000 (Peqlab, Erlangen, Germany). RNA integrity was determined on the Experion Automated Electrophoresis System using the Experion RNA StdSens Analysis Kit (Bio-Rad). After passing quality controls RNA samples were preprocessed with the 3' IVT Express Kit (Affymetrix, Santa Clara, CA, USA) according to the manufacturer's instructions. Samples were hybridized on a GeneChip HT MG-430 PM 16-Array Plate using the GeneTitan MC Instrument (Affymetrix) and the GeneTitan Hybridization, Wash, and Stain Kit (Affymetrix). CEL files were processed for further data analysis by the GeneChip Command Console Software (AGCC, version 3.2.4, Affymetrix). Robust multiarray average (RMA) normalization was carried out with the Expression Console Software (version 1.2.1, Affymetrix). Array data can be found at (http://www.ncbi.nlm.nih.gov/geo/query/acc.cgi?acc=GSE85381), under the accession number GSE85381.

HepaRG cell culture and APAP treatment:

HepRG were cultured as described previously [2]. Cells (HepaRG or HepG2) were treated with different concentrations of APAP (Sigma-Aldrich) diluted in cell culture medium for 24h and used for RNA isolation or generation of protein lysates.

Autophagic Flux Assay:

Autophagic flux was measured by LC3-II turnover using Western blot analysis for LC3-II and p62 in pcIneo- control or pcIneo-KLF6-over-expressing HepG2 cells treated with 100µM chloroquine diphosphate or corresponding vehicle for 24h.

KLF6-binding DNA elements in ChIP-seq:

ChiIP-Seq data for KLF6 antibody assayed in HepG2 cells were obtained from the NIH Encyclopedia of DNA Elements (ENCODE) project portal website (www.encodeproject.org) (accession numbers: KLF6, ENCSR757EKM; control, ENCSR239QGH). Significant KLF6-binding peaks were called based on either relaxed, conservative, or optimal criteria (see www.encodeproject.org/chip-seq for methodological details). ChIP-seq reads were mapped onto human reference genome (assembly GRCh38) and visualized by Integrative Genomics Viewer ver. 2.3.91 (www.broadinstitute.org/igv).

Supplementary Figures:

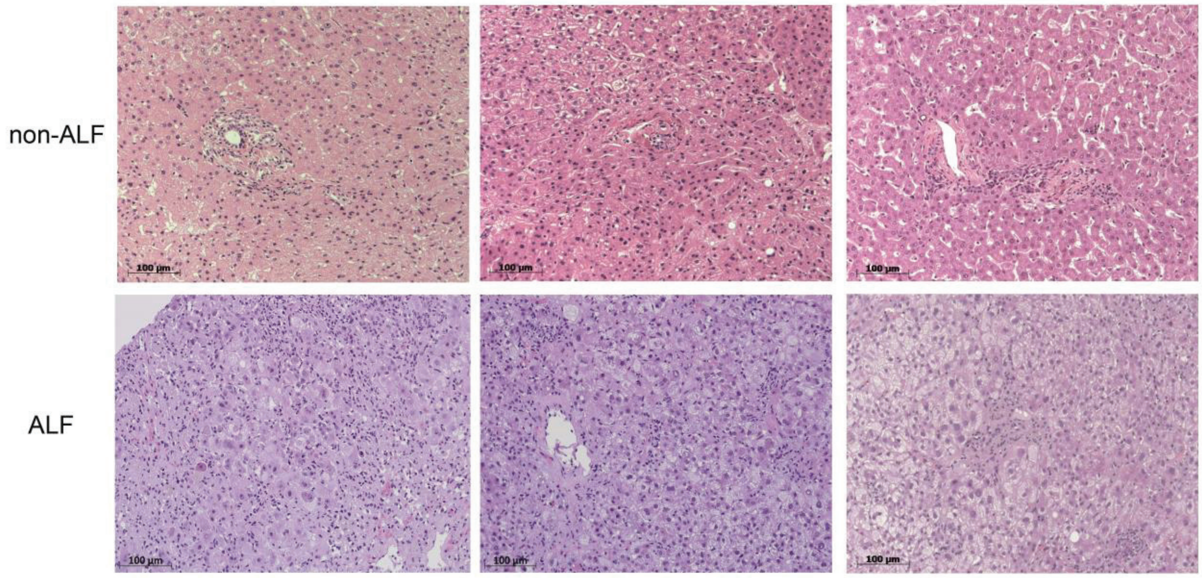

Supplementary Figure S1: H&E staining of liver sections from patients with acute liver failure (ALF). Shown are representative tissue sections of patients with drug-induced acute liver failure (ALF) or non-acute liver injury (non-ALF) patients stained with H&E (20-fold magnification).

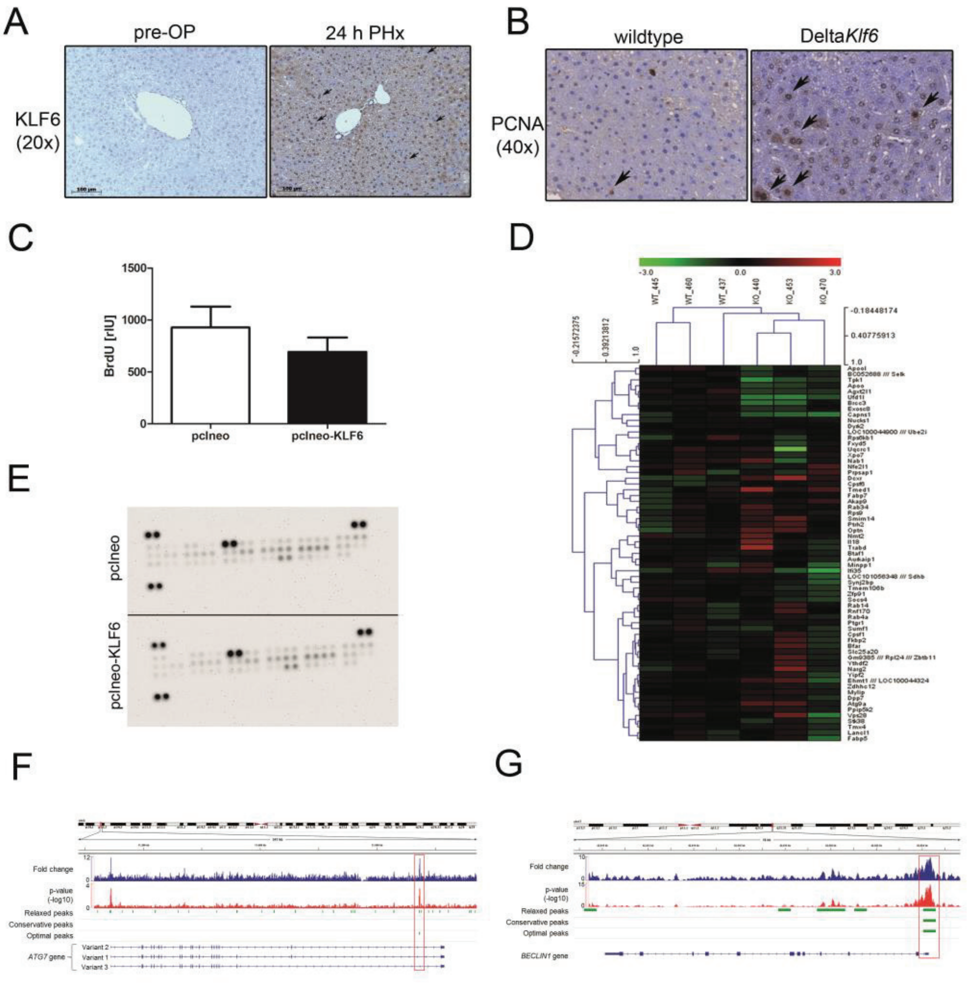

Supplementary Figure S2: KLF6 affects liver regeneration and autophagy after partial hepatectomy (PHx). Representative images of (A) KLF6 immunohistochemistry in liver tissue of wt mice before (pre-OP) and 24h after partial hepatectomy (PHx, 20-fold magnification) and (B) PCNA staining of liver tissue from wt or Delta*Klf6* mice 12h after PHx (40-fold magnification). (C) BrdU proliferation Assay was performed in HepG2 cells transiently transfected with an empty control vector (pcIneo) or a KLF6 expression vector (pcIneo-KLF6). (D) Heatmap from Affymetrix gene array includes all regulated genes in liver tissue whether from wt or from Delta*Klf6* mice 12h after PHx. (E) Proteome Profiler Antibody/human Apoptosis Array was performed using cell lysates from HepG2 (pcIneo) and KLF6-over-expressing HepG2 (pcIneo-KLF6) cells. KLF6-binding genomic regions encoding *ATG7* (F) and *BECLIN1* (G) by ChIP-seq in HepG2 cells. Fold change of KLF6-binding peaks over control and peak calls at three different criteria are shown and highlighted by red boxes (see Supplementary methods for details).

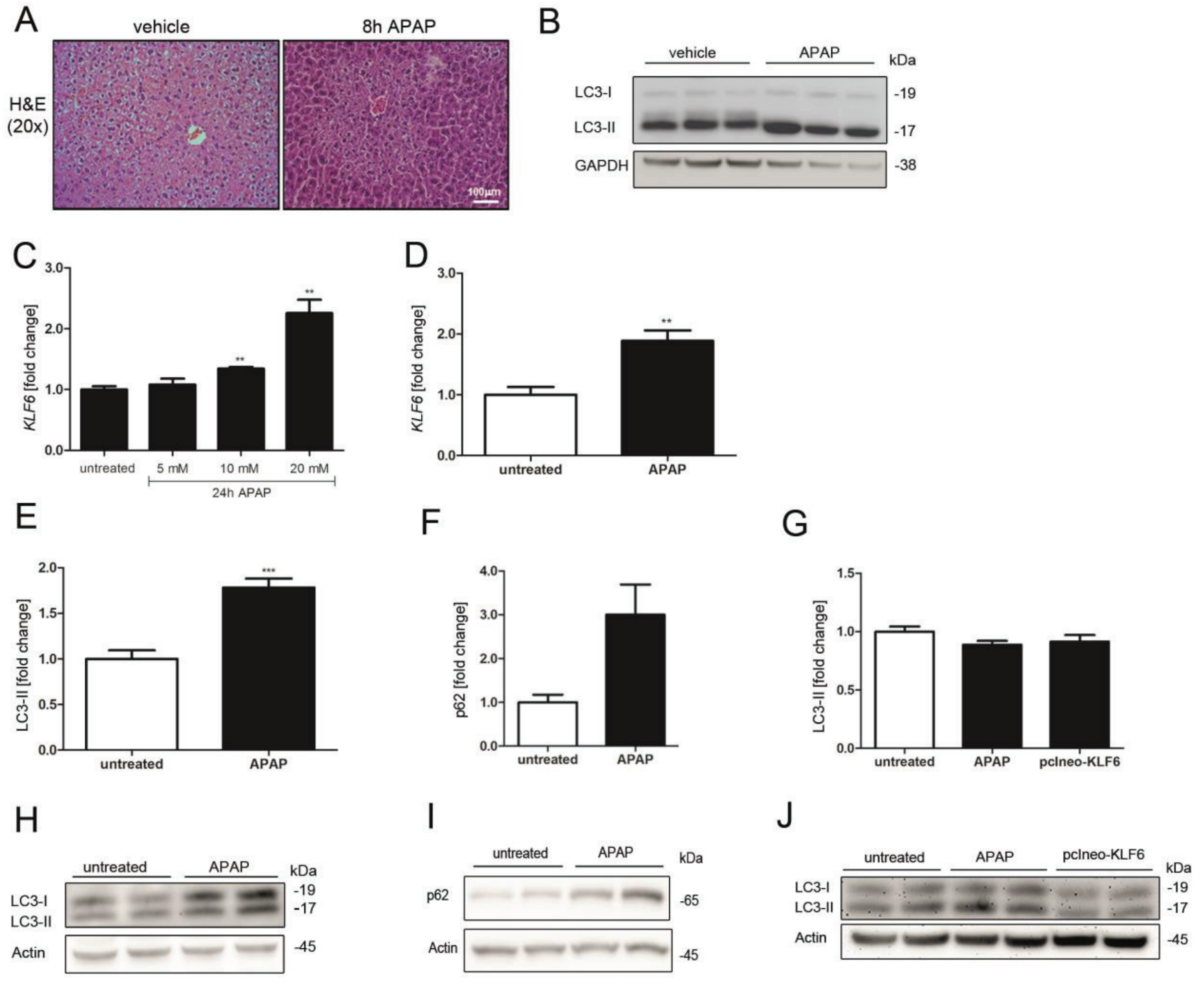

Supplementary Figure S3: Acute liver injury induces KLF6-expression and affects autophagy induction. Representative tissue sections stained with H&E (A) of mouse liver tissue treated with acetaminophen or vehicle (500mg/kg liver) for 8h are shown (20-fold magnification). Representative Western blot images of mice treated with APAP show an induction of LC3-II levels in liver tissue of these animals (B) *KLF6*-expression levels were measured by qRT-PCR in (C) HepaRG cells treated whether with 5mM, 10mM or 20mM APAP for 24h and in (D) HepG2 cells treated with 5mM APAP for 24h (n=3). Densitometric quantification and representative Western blot images for LC3-II and Actin as loading control are shown for HepG2 cells treated with 5mM APAP for 24h are in panels E and H. Western blot images for p62 and Actin (I) as loading control as well as densitometric quantification (F) are shown for untreated HepG2 cells or, HepG2 treated with 5 mM APAP for 24h. Representative Western blot images of LC3-II-levels and loading control Actin in p53 deficient HepG2-303 after treatment with 5mM APAP for 24h and in KLF6-over-expressing cells (J), and quantified by densitometry normalized to Actin levels (G).

Supplementary Tables

Supplementary Table S1: Data from patients (ALF and non-ALF) used for immunohistochemistry

	**Acute liver failure (n=15)#**		**Non-acute liver injury (n=6)**
	**Drug-induced ALF(n=7)**	**AIH (n=8)**	
**Age [years]**	42.86 ± 5.33	42.63 ± 3.46	48.5 ± 5.03
**Gender [f/m]**	7/0	5/3	3/3
**ALT [U/l]**	1978.00 ± 648.30*	2066.00 ± 411.60**	17.83 ± 4.05
**AST [U/l]**	1763.00 ± 559.70*	1558.00 ± 317.60**	20.17 ± 2.20
**Gamma glutamyltransferase [U/l]**	143.60 ± 57.97	243.00 ± 56.80**	26.17 ± 5.66
**Total bilirubin [mg/dl]**	12.57 ± 3.30*	16.00 ± 1.07***	0.47 ± 0.11
**Creatinine [mg/dl]**	0.86 ± 0.24	0.65 ± 0.13	1.40 ± 0.62
**INR**	1.99 ± 0.43	1.7 ± 0.14***	0.96 ± 0.04
**KLF6 positive cells (IHC)/visual field [%]**	50.80 ± 19.14*	66.01 ± 10.42 ***	0.58 ± 0.22

#Patients: 8 ALF patients with autoimmune hepatitis (AIH), 7 ALF patients based on drug toxicity (2x acetaminophen, 2x ibuprofen, 2x flupirtine, 1x valproic acid; biopsies were taken 2-23 days (mean 8.6 days) after occurrence of jaundice. IHC (immunohistochemistry) quantification shows mean ± SEM of KLF6 positive hepatocytes per total cells quantified of 4 visual fields per patient (40-fold magnification). *** $p<0.001$; ** $p< 0.01$; * $p<0.05$ versus non-acute liver injury.

Supplementary Table S2: Quantification of autophagosome-positive cells visualized via TEM (transmission electron microscopy) or Autophagy Tandem Sensor RFP-GFP-LC3B assay in HepG2 cells transiently transfected with empty vector (pcIneo) or *KLF6* expression vector (pcIneo-KLF6).

	**pcIneo**	**pcIneo-KLF6**	**pcIneo + Rapamycin**
**TEM (% autophagosome-positive cells/visual field)**	**4.4 ± 1.0**	**23.5 ± 2.5***	**39.0 ± 3.0***
**Tandem Assay (% autophagosome-positive cells/visual field)**	**2.7 ± 0.7**	**15.7 ± 2.1****	**28.4 ± 3.1****

* $p<0.05$; ** $p<0.01$ (vs. pcIneo)

Supplementary Table S3: mRNA expression levels of apoptosis-related genes in HepG2 cells transiently transfected with empty vector (pcIneo) or *KLF6* expression vector (pcIneo-KLF6)

Gene	HepG2 (pcIneo) (fold change)	HepG2 (pcIneo-KLF6) (fold change)
**BAX**	1.00 ± 0.04	0.93 ± 0.05
**BID**	1.00 ± 0.07	0.88 ± 0.04
**BCL-2**	1.00 ± 0.28	0.93 ± 0.11
**P21/ CDKN1A**	1.00 ± 0.02	0.76 ± 0.05**

** $p<0.01$ (pcIneo vs. pcIneo-KLF6)

Supplementary Table S4: Oligonucleotide sequences utilized for qRT-PCR and ChIP

	**forward**	**reverse**
KLF6/Klf6 (human/mouse)	5’-CGGACGCACACAGGAGAAAA-3’	5’-CGGTGTGCTTTCGGAAGTG-3’
Atg7 (mouse)	5’-TGCCTCACCAGATCCGGGGTT-3‘	5‘-CGCTAGGAAGGTGAATCCTTCTCG-3‘
BAX (human)	5’-TCTGACGGCAACTTCAACTG-3’	5’-GGAGGAAGTCCAATGTCCAG-3’
BCL-2 (human)	5’-GGATAACGGAGGCTGGGATG-	5‘-CAGGGCCAAACTGAGCAGA-3‘

	3’	
Beclin1 (mouse)	5‘-TGCCTCACCAGATCCGGGGTT-3‘	5‘-CGCTAGGAAGGTGAATCCTTCTCG-3‘
BID (human)	5‘-ACTCCCGCTTGGGAAGAATA-3‘	5‘-GAGGGATGCTACGGTCCAT-3‘
HPRT (human)	5‘GACCAGTCAACAGGGGACAT-3‘	5‘-CTTGCGACCTTGACCATCTT-3‘
P21 (human)	5‘-GACTCTCAGGGTCGAAAACG-3‘	5‘-GGATTAGGGCTTCCTCTTGG-3‘
Sdha (mouse)	5’-GCGGCTTTCACTTCTCTGTT-3’	5’-TCCACCACTGGGTATTGAGT- 3’
ATG7 (ChIP, human)	5’-AGACGCTCTCCATCGCTTC-3’	5’-CTCACTTACCGCCGCTCAA-3’
BECLIN1 (ChIP, human)	5’-CTTGGCTCCTACACTTCCCG-3’	5’-GGCCTCCAGAACTACCATCG-3‘

Supplementary References:

1. Kohl, M. *et al.* A practical data processing workflow for multi-OMICS projects. *Biochim. Biophys. Acta* 1844, 52–62 (2014).
2. Nelson, L. J. *et al.* Human Hepatic HepaRG Cells Maintain an Organotypic Phenotype with High Intrinsic CYP450 Activity/Metabolism and Significantly Outperform Standard HepG2/C3A Cells for Pharmaceutical and Therapeutic Applications. *Basic Clin. Pharmacol. Toxicol.* (2016). doi:10.1111/bcpt.12631

LC3 Western blot full image (cutout shown in Figure 2E, pre-OP LC3 wildtype vs. delta*Klf6*)

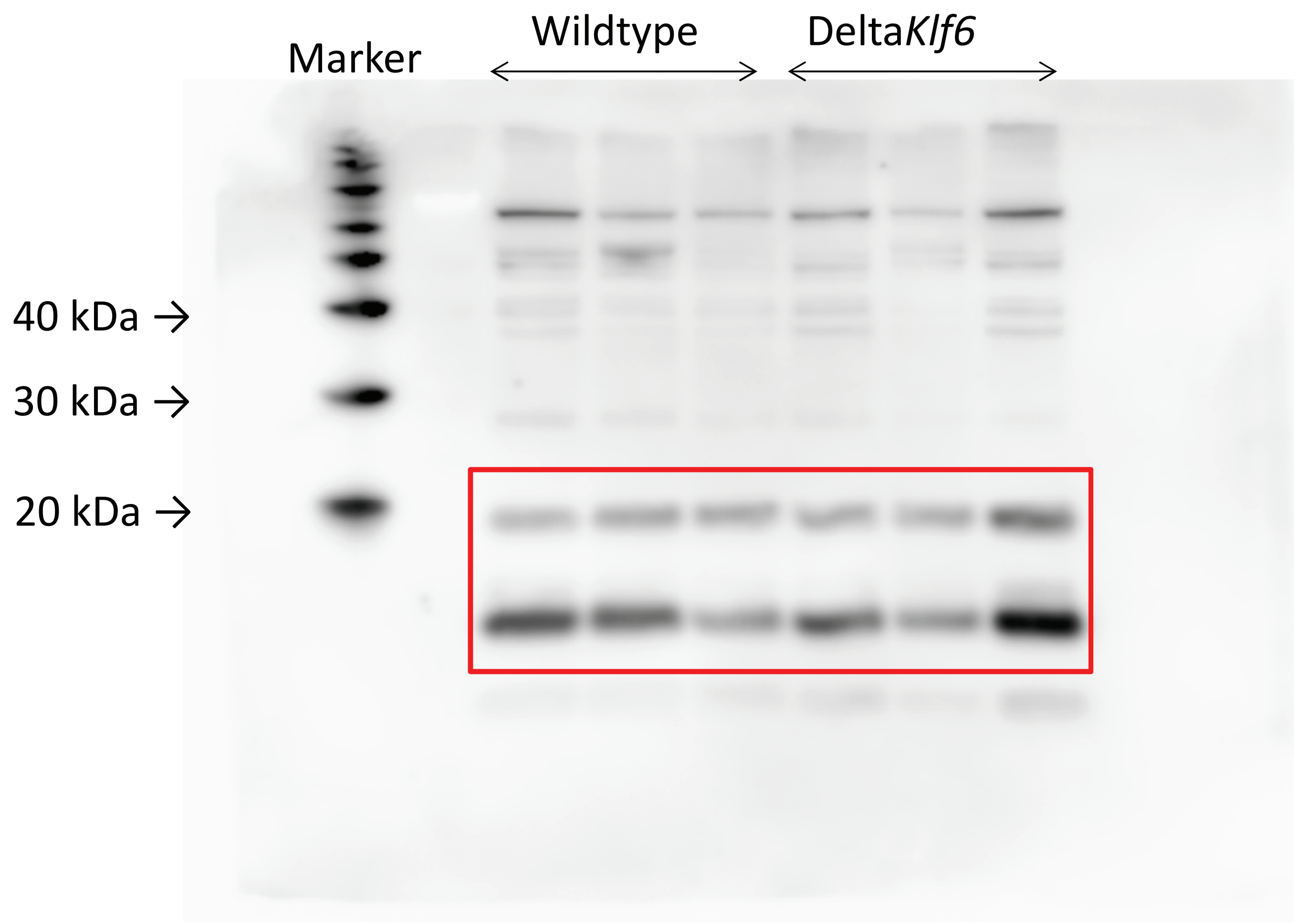

Actin Western blot full image (cutout shown in Figure 2E, LC3 pre-OP wildtype vs. Delta*Klf6*)

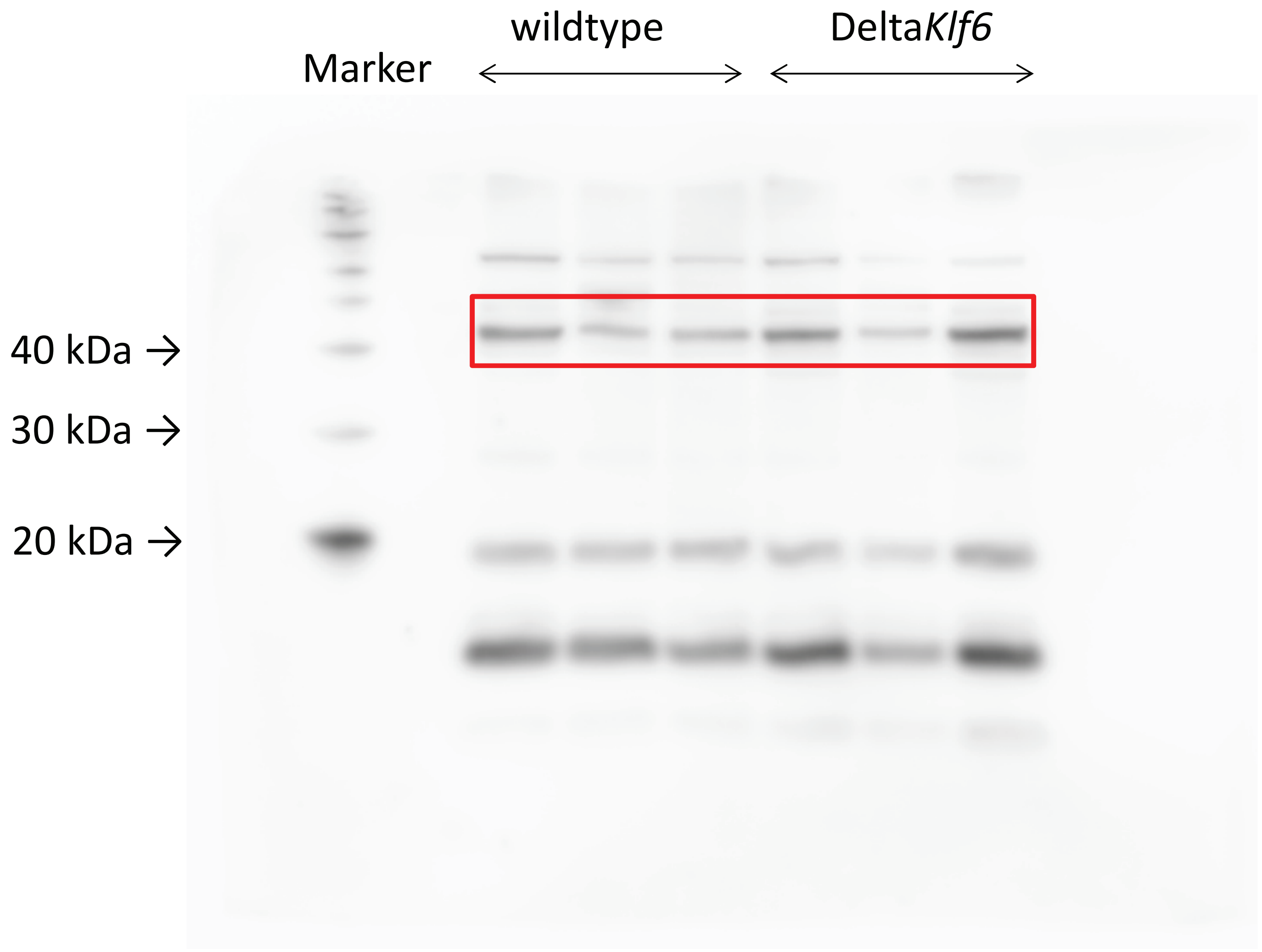

LC3 Western blot full image (cutout shown in Figure 2E, postPHx)

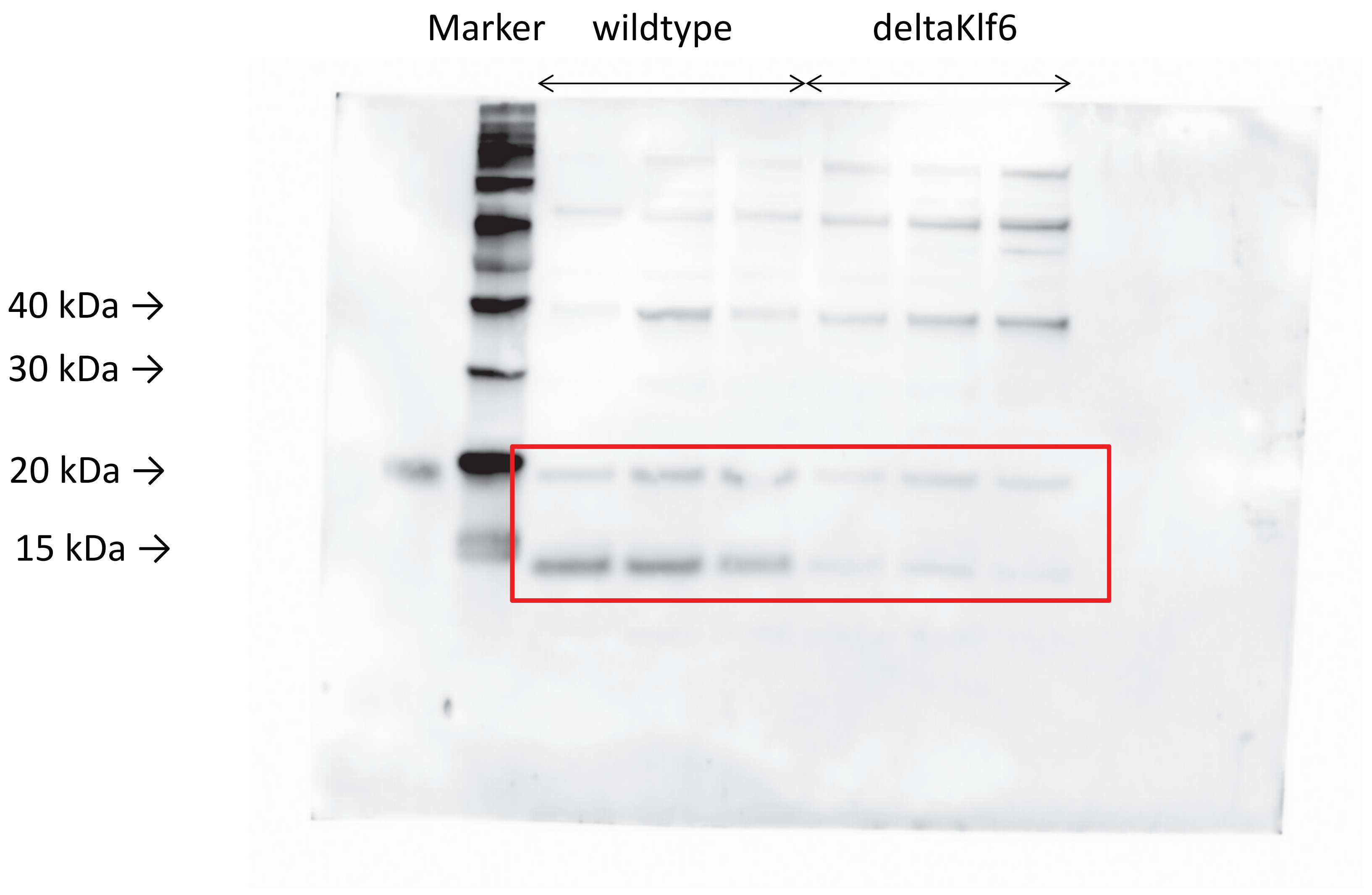

Actin Western blot full image (cutout shown in Figure 2D)

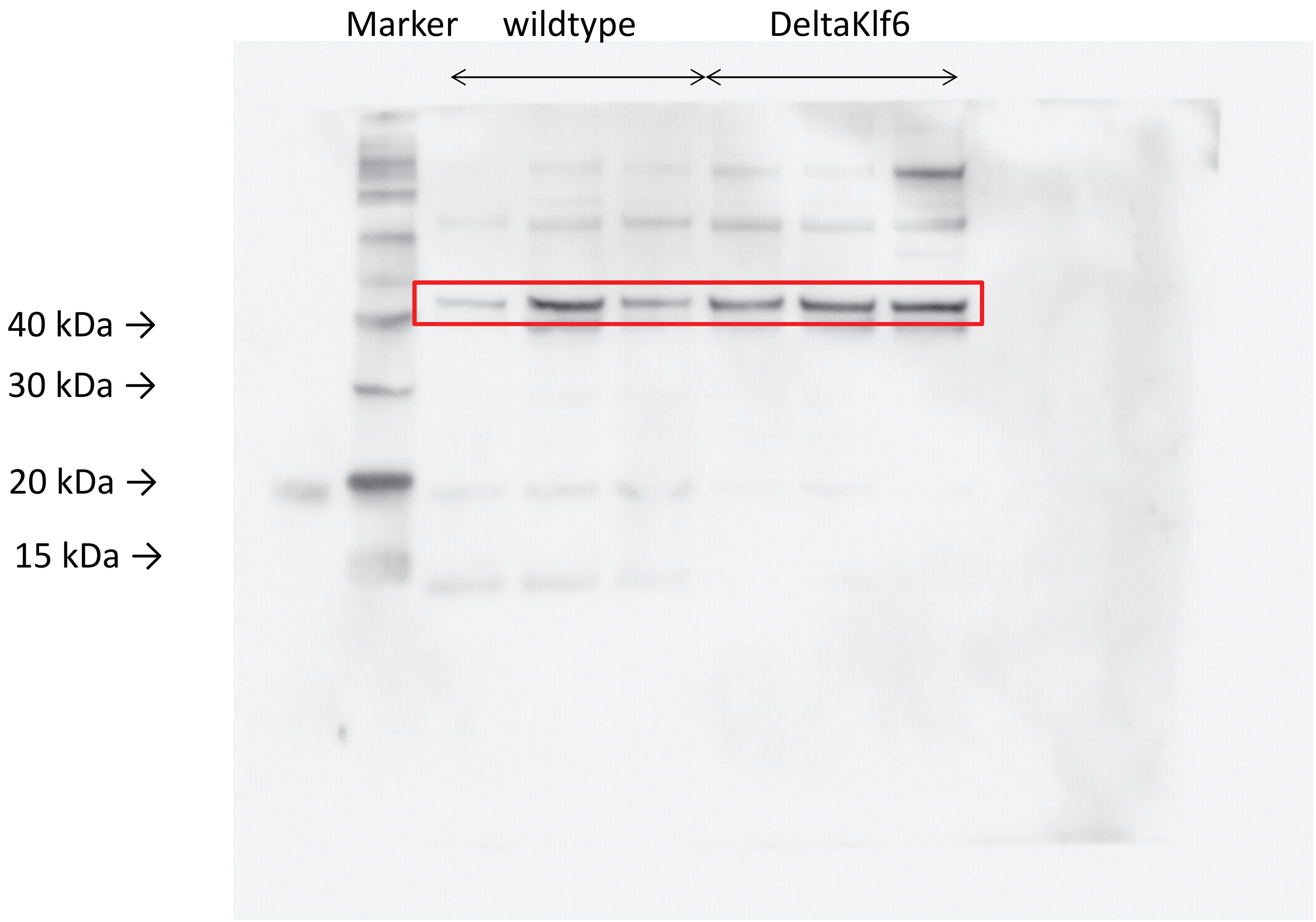

LC3/Actin Western blot full image (cutout shown in Figure 3E, wildtype)

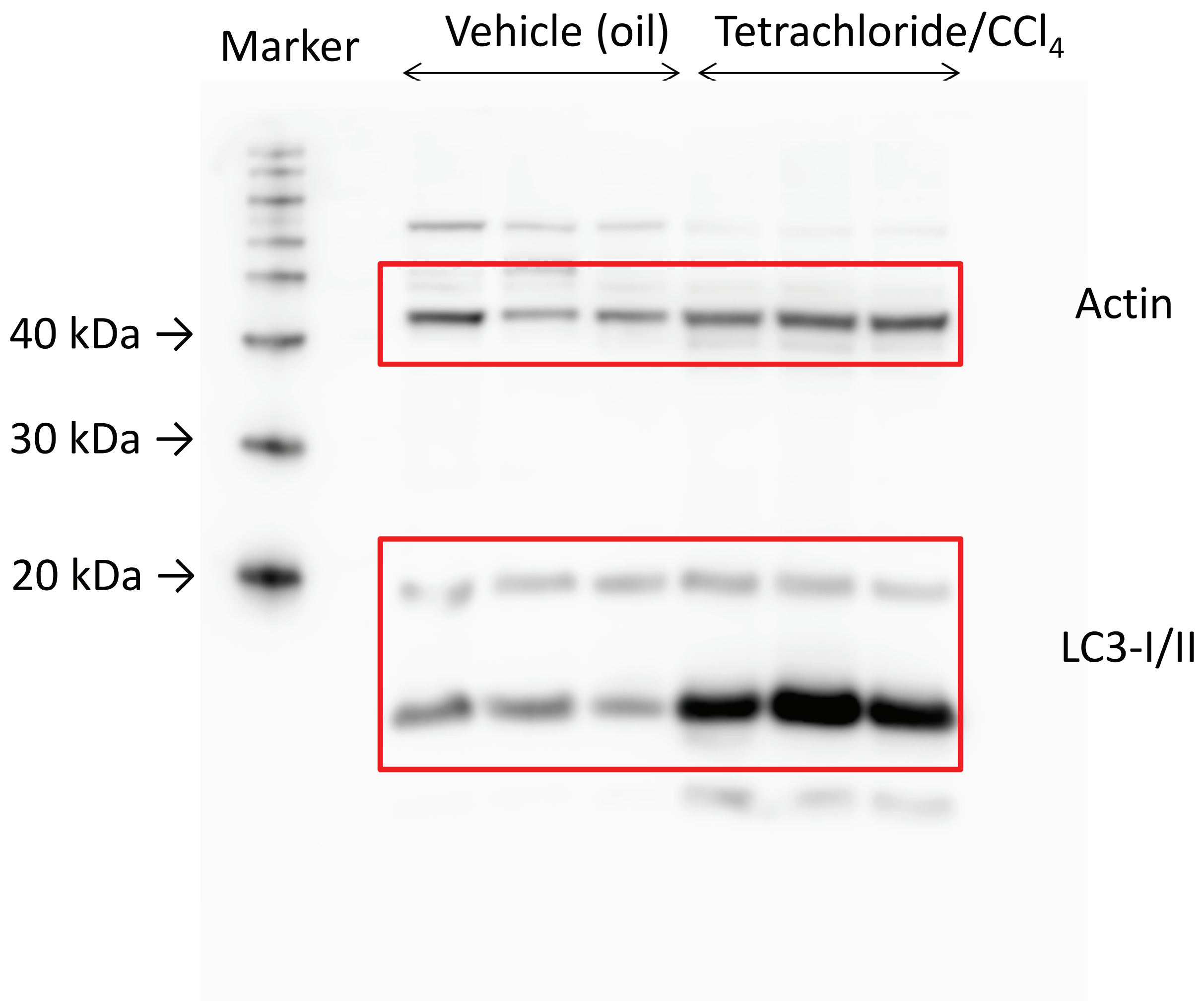

LC3 Western blot full image (cutout shown in Figure 3E, delta*Klf6*)

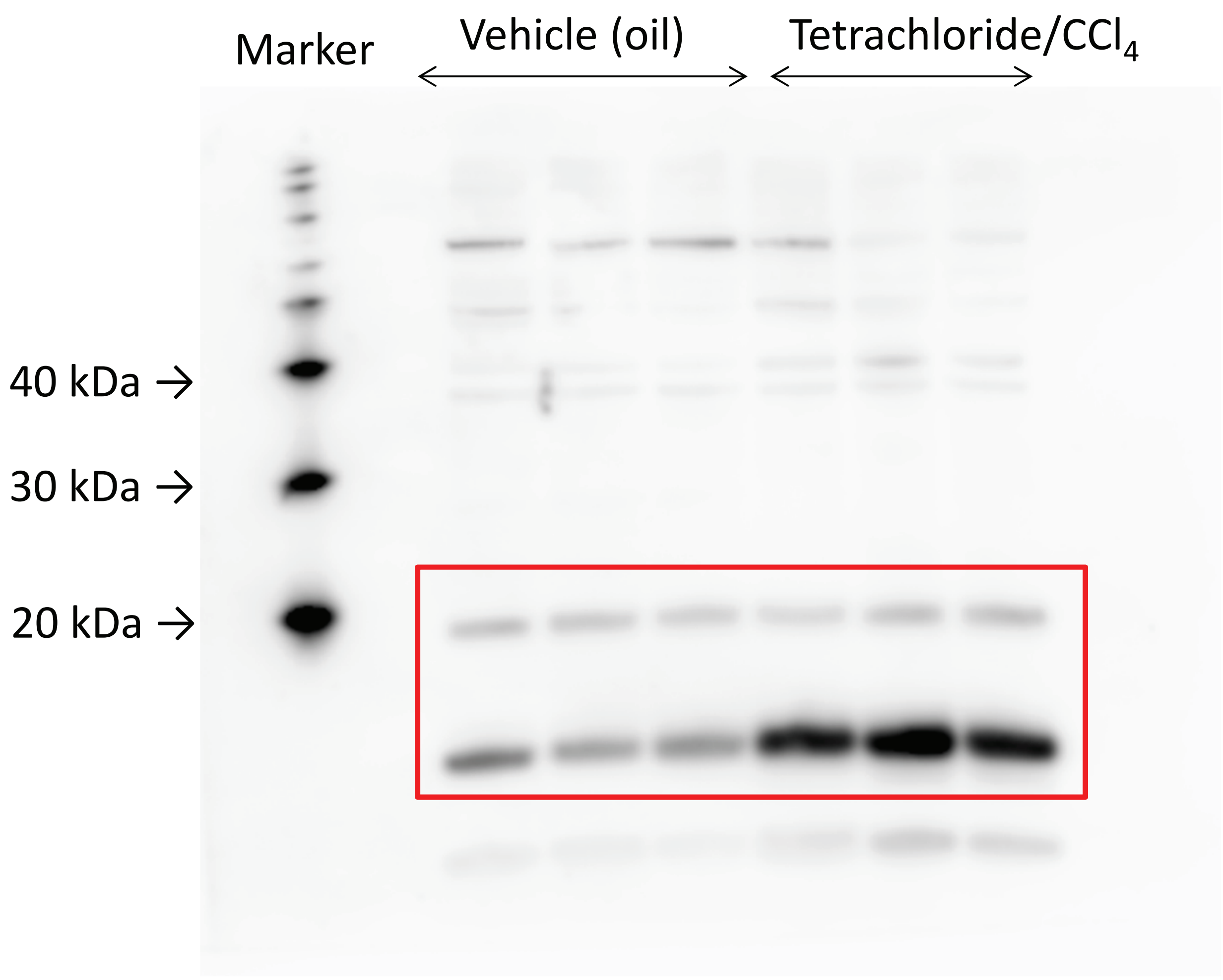

Actin Western blot full image (cutout shown in Figure 3E, delta*Klf6*)

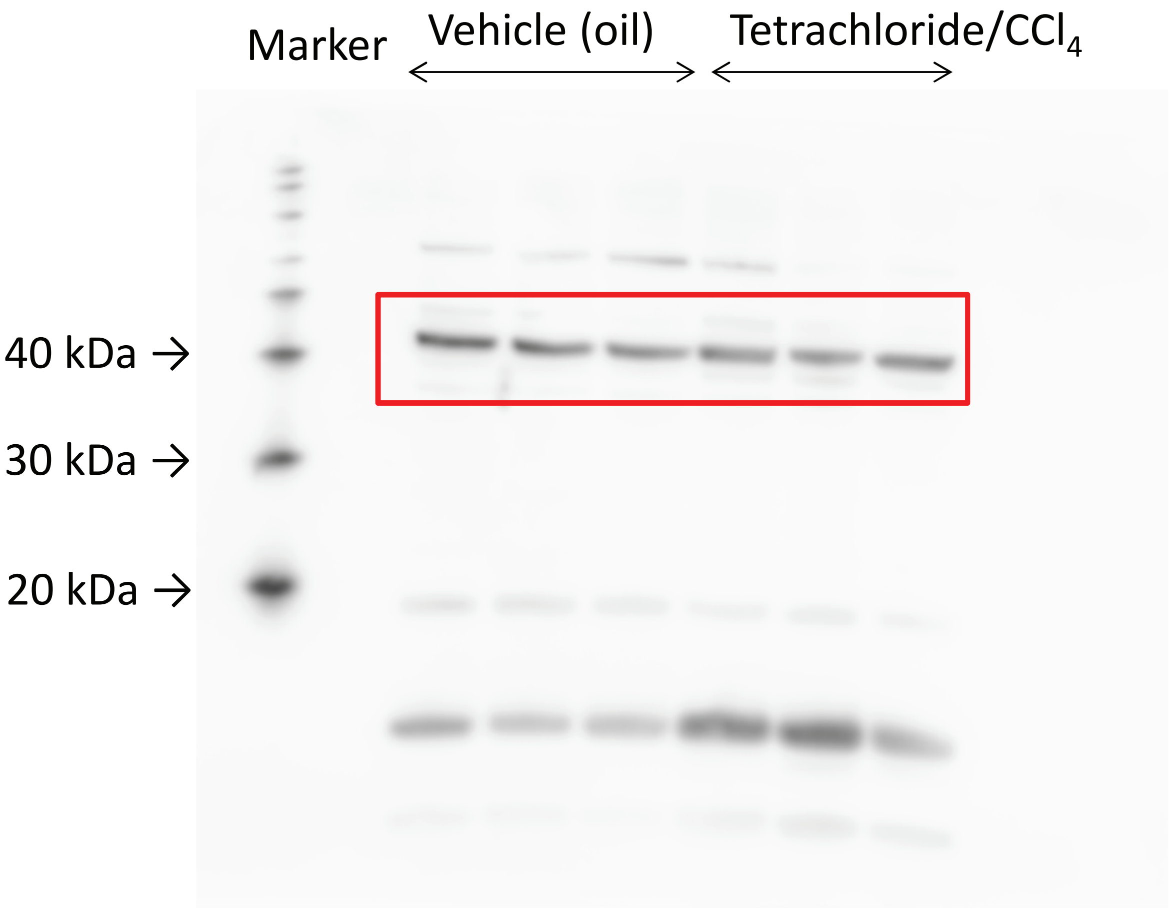

KLF6 Western blot full image (cutout shown in Figure 4B)

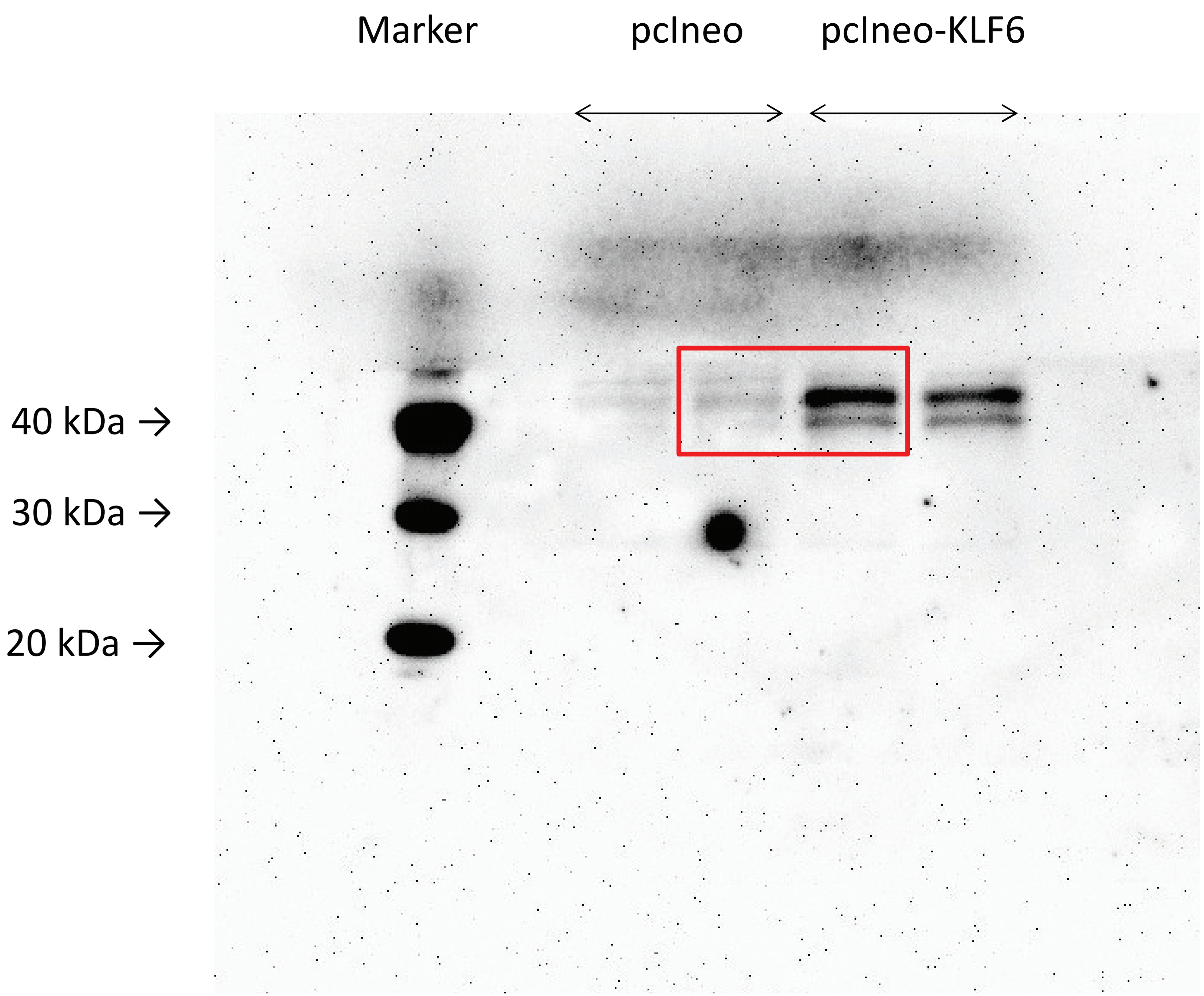

GAPDH Western blot full image (cutout shown in Figure 4B)

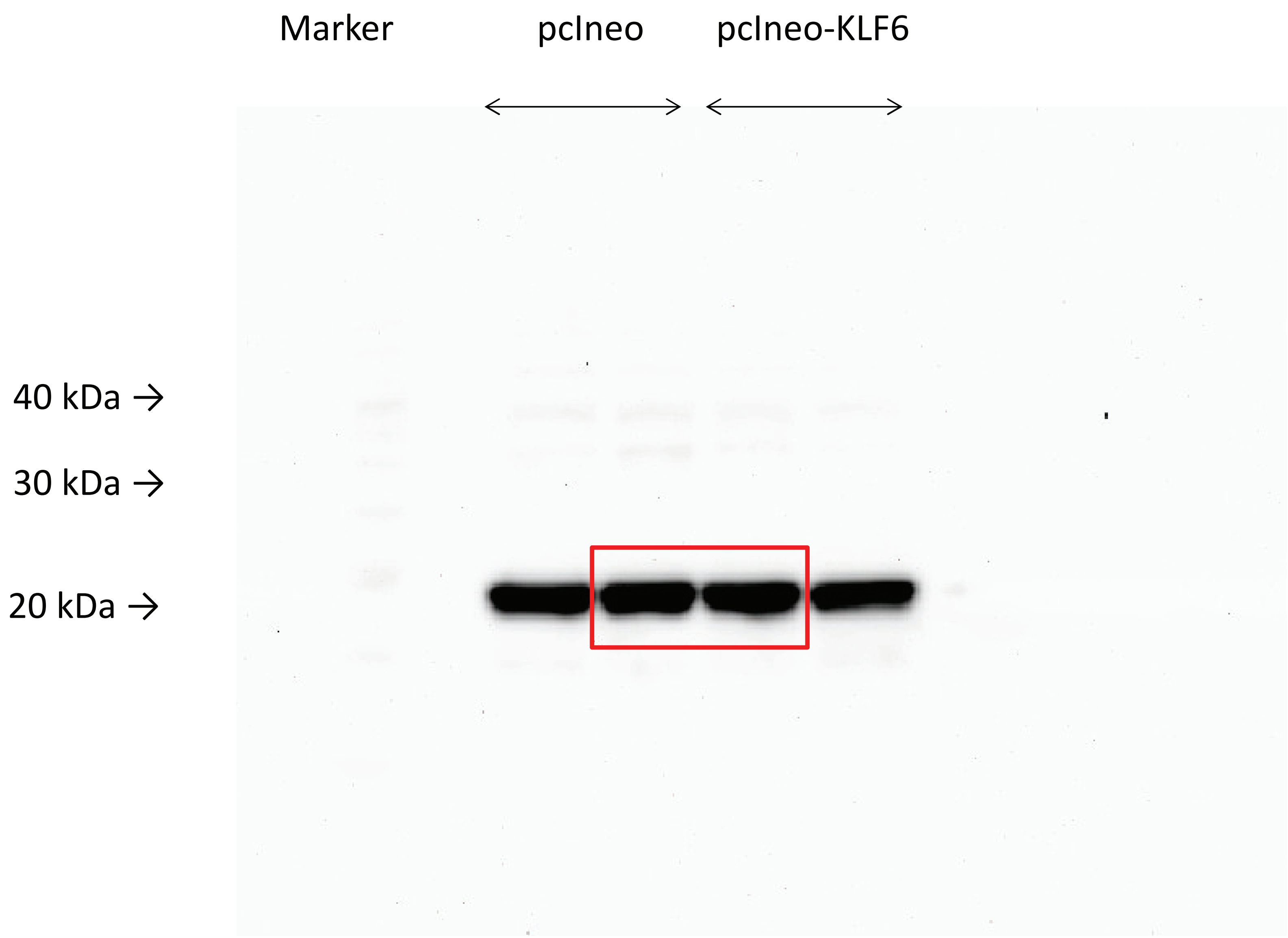

p62 Western blot full image (cutout shown in Figure 4C)

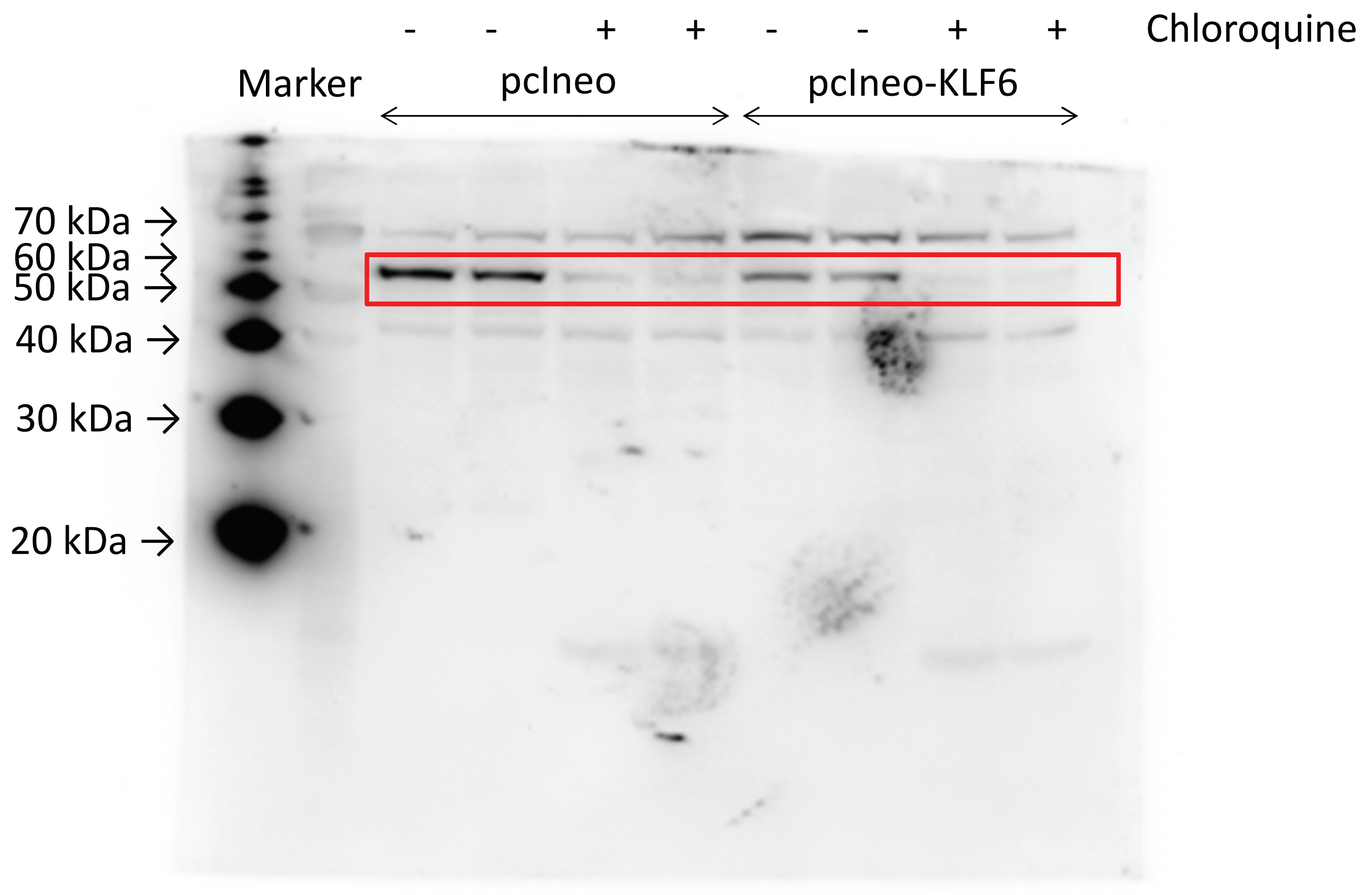

LC3 Western blot full image (cutout shown in Figure 4C)

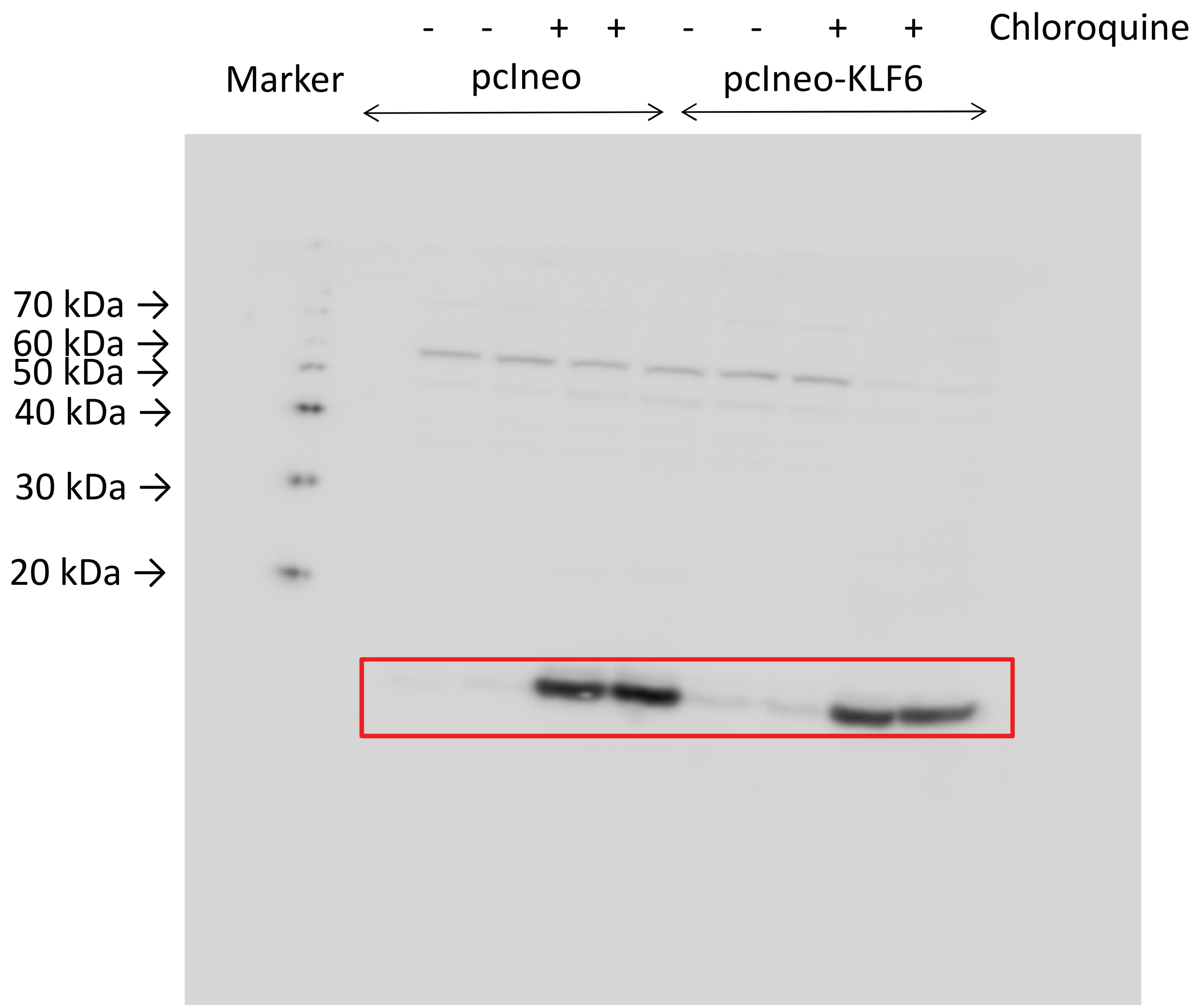

GAPDH Western blot full image (cutout shown in Figure 4C)

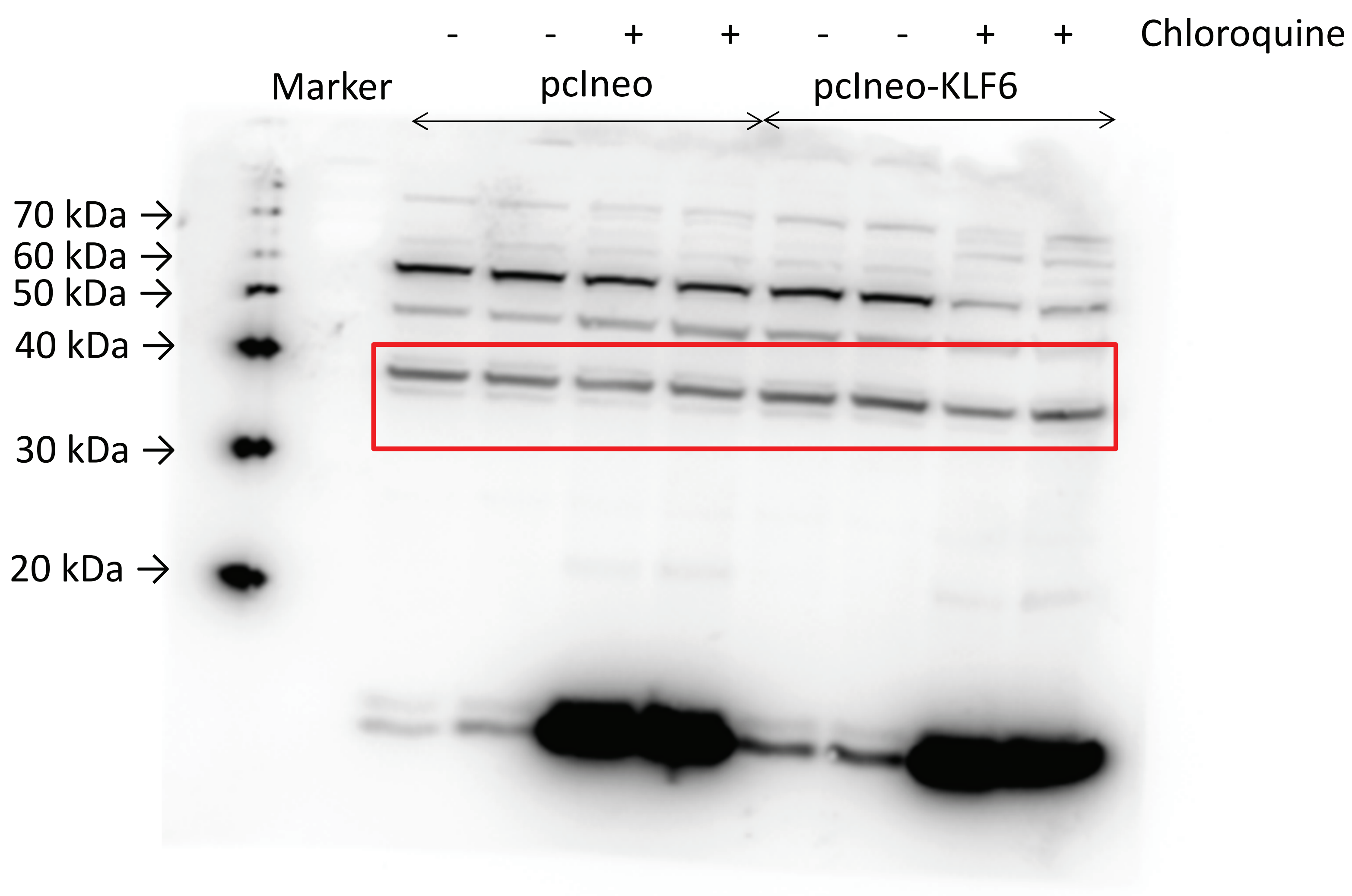

ChIP PCR result-agarose gel full image (cutout shown in Figure 4G)

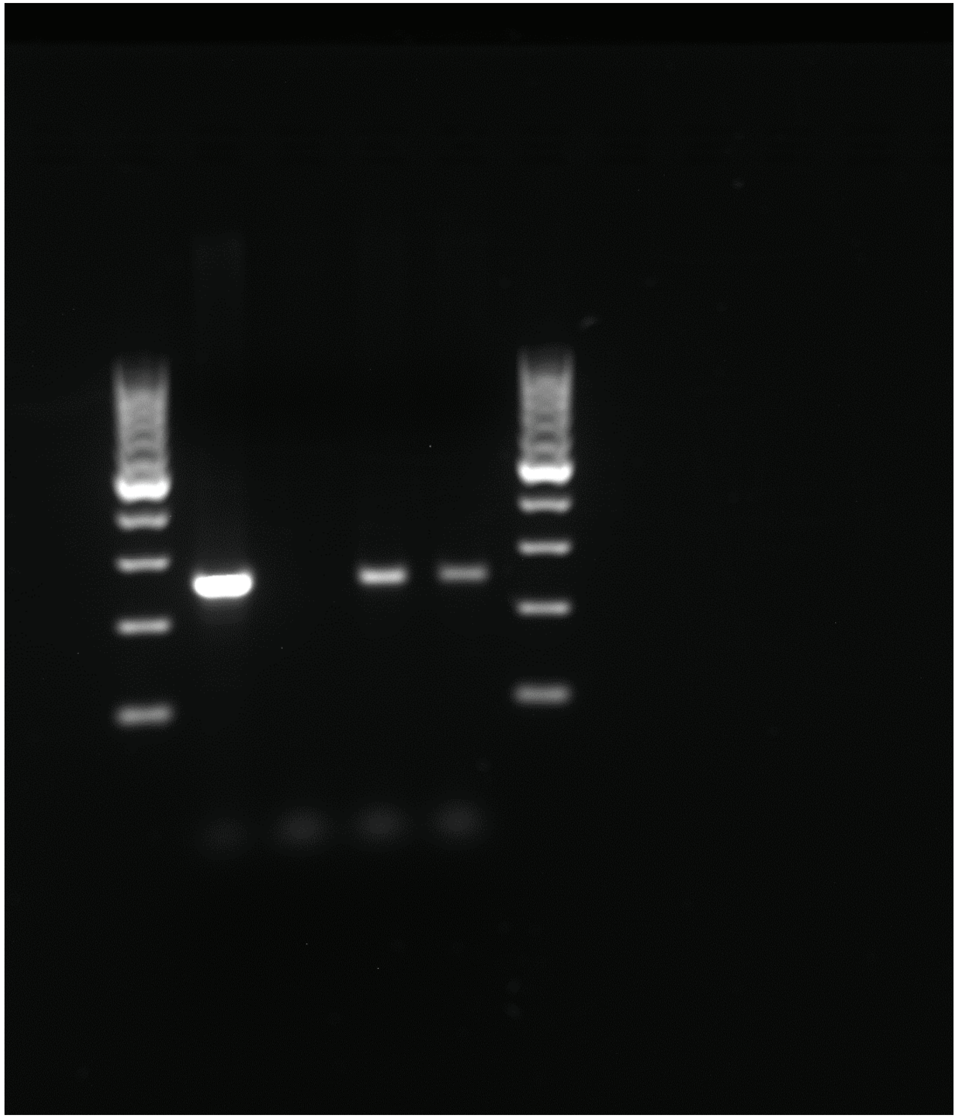

ChIP PCR result-agarose gel full image (cutout shown in Figure 4G)

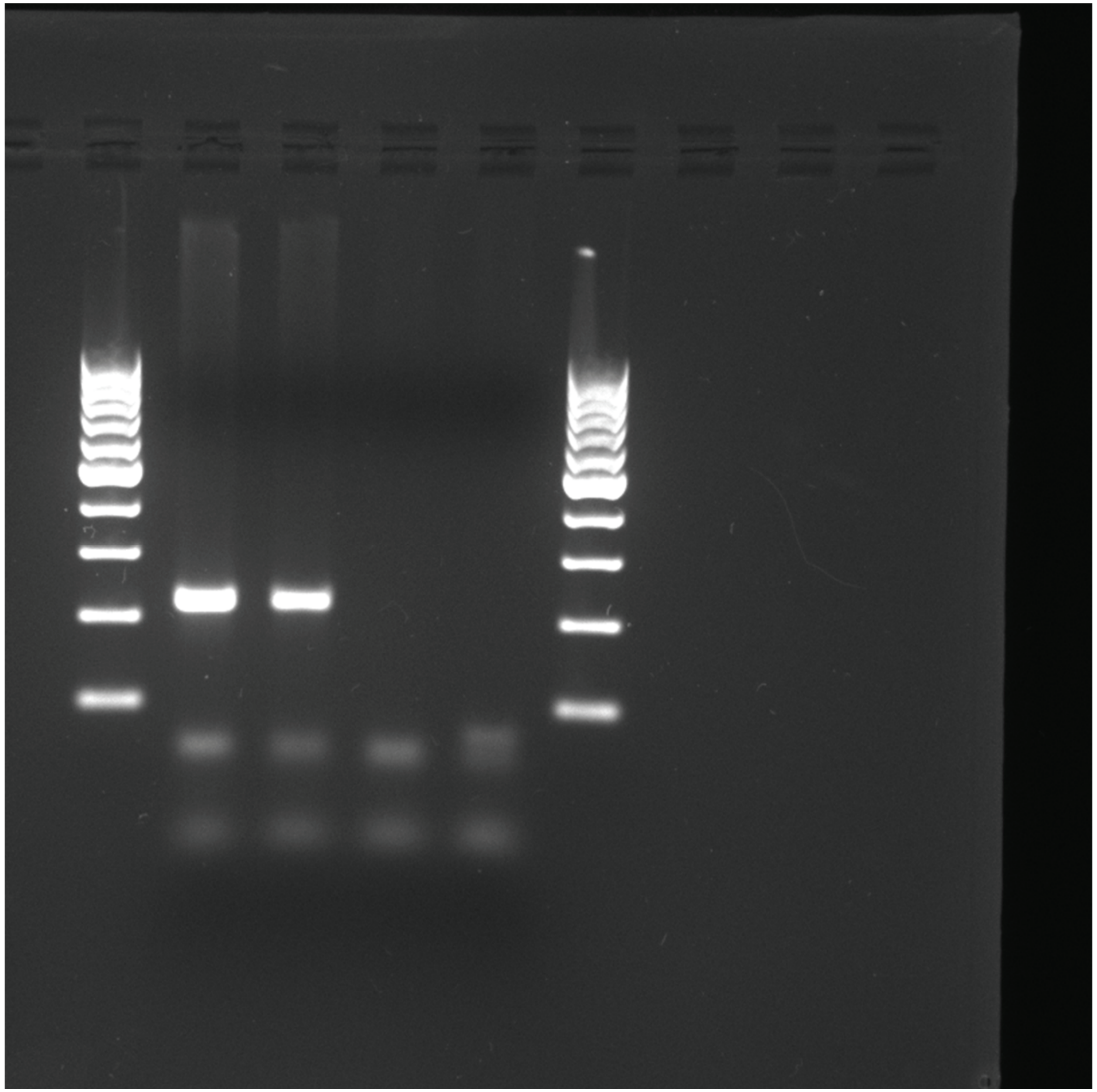

Supplement: Supplementary file 1 — Supplementary Information [file 41598_2017_8680_MOESM1_ESM.pdf]
